# Gene transcriptional profiling of osmoregulatory stress response in salmonids: Development and assessment of high-throughput multispecies assays

**DOI:** 10.1371/journal.pone.0360513

**Published:** 2026-10-09

**Authors:** Nicolas Tugui, Shahinur S. Islam, Daniel D. Heath

**Affiliations:** 1 Department of Biology, University of Windsor, Ontario, Canada; 2 Great Lakes Institute for Environmental Research, University of Windsor, Ontario, Canada; 3 School of Biological Sciences, Washington State University, Pullman, Washington, United States of America; University of Iceland, ICELAND

## Abstract

Osmoregulatory stress is a critical factor in the welfare and performance of salmonids under captive rearing for aquaculture and conservation, and characterizing cellular responses to that stress may foster better husbandry. One approach for early detection of osmotic stress is gene transcriptional profiling, where a suite of qPCR assays designed for related-function genes is used to determine patterns of transcriptional responses to stress. This study describes the development, assessment and application of 28 Taqman qPCR assays for use on the OpenArray® platform as an osmoregulatory stress transcriptional profiling (OSTP) chip. The assays include key genes involved in osmoregulation (twelve), stress response (four), immune function (five), growth/metabolism (four) and endogenous controls (three). Here, using gill and liver tissues from eight salmonid species across four genera, the OSTP chip’s performance was evaluated. While results showed both tissue- and species-specific transcriptional differences, the OSTP chip has considerable utility for characterizing molecular stress responses. As a test application of the OSTP chip, we performed a 24-hour saltwater challenge with Chinook salmon and applied the chip to control and challenged fish. This test confirmed the OSTP chip’s capacity to detect acute osmotic stress-induced molecular genetic signatures. This OSTP chip will be useful for aquaculture practices by fostering better husbandry as well as providing a valuable tool for future studies of the osmoregulatory response of salmonid fishes to environmental stress.

## 1. Introduction

Osmoregulation in anadromous salmonids is a well-explored aspect of their physiology [1], with extensive research targeting their remarkable ability to adapt to a large range of salinity levels [2]. These species generally display exhibit complex mechanisms for regulating ion balance and water uptake [3], which is key for maintaining osmotic balance while transitioning between freshwater and marine environments through their life cycle [4]. Specifically, juveniles adapt to seawater before migrating to the ocean (smolting; [5]) and upon returning to spawn, salmonids undergo the reverse process of freshwater adaptation [6]. This adaptability involves complex interactions between ion transport, hormone regulation, and cellular osmoregulatory mechanisms [7]. Through decades of study, researchers have characterized key genes and pathways involved in osmoregulation [8], thus providing a solid foundation for molecular genetic analyses of the salmonid osmoregulatory coping mechanisms [9].

Our extensive knowledge of the dynamics of osmoregulation in wild and captive populations of salmonids underscores that the processes can differ depending on environmental context [10], since the fish experience different stressors in the wild versus captivity [11]. One obvious example is freshwater rearing, where the fish are maintained in freshwater for their entire life cycle, which is increasingly common in commercial aquaculture [12]. In enhancement hatchery programs, salmonids are reared in hatcheries and later released into the wild at the smolt stage [13]. As the goal of those programs is to improve the conservation status and productivity of wild salmon stocks, smolt quality and fitness are of high priority [14]. This emphasizes the need for a quick and reliable advanced molecular genetic method to quantify and monitor salmonid osmoregulatory stress in captive and wild populations to enhance captive rearing and management, and conservation protocols, and ultimately, improve animal welfare.

In response to the challenges posed by stress in captive and wild environments, fish culturists, conservationists, and fishery managers are increasingly turning to molecular genetic tools for fish health monitoring [15]. Transcriptional profiling, which is the measurement of transcription of multiple related-function genes, allows broad assessment of candidate gene network responses to environmental stimuli [16]. This approach is powerful because different gene expression pathways may be triggered depending on the genetic background of the fish, and many specific gene functions remain uncertain in non-model organisms such as most fish species [17]. By measuring the transcription patterns of genes involved in osmoregulatory stress, researchers can identify the molecular signatures indicative of stress before fish health is compromised [18]. Such a proactive approach enables fish culturists to implement targeted interventions to modify rearing conditions to alleviate osmoregulatory stress and promote fish welfare [19]. Furthermore, transcriptional profiling provides valuable insights into the adaptive response of salmonids to stress, shedding light on the molecular mechanisms underlying this form of plasticity [20].

Here, we described the development and assessment of a panel of gene transcription Taqman^TM^ qPCR assays designed for the OpenArray® nanofluidic platform, targeting multiple genes associated with osmoregulatory and related stress responses in salmonid fishes. The panel included assays for osmoregulatory function (twelve), stress response (four), immune function (five), growth and metabolism (four) and three endogenous control genes. This advanced technology offers rapid, high-throughput, and low-cost analyses, providing a valuable tool for monitoring fish health and coping mechanisms and fostering better husbandry [21]. However, while gene expression assays can provide valuable molecular data, they may not always be the fastest approach compared to more immediate physiological markers such as plasma chloride levels. We explored the utility of the OSTP chip by applying it to a controlled experimental acute stress test (24-hour saltwater challenge). The OSTP chip not only advances our basic understanding of the osmoregulatory stress response but also has significant implications for improving aquaculture practices, paving the way for more effective stress management and healthier fish. Future developments may include expanding the assay panel to cover additional stress-related pathways, coupled with commercialization of the protocols for “fee for service” agreements.

## 2. Methods

### 2.1. Candidate gene selection

In selecting candidate genes for the OSTP chip, we targeted gene function categories relevant to osmoregulatory stress, ensuring coverage of key physiological responses. We included three endogenous control genes: Elongation factor 1-alpha (*EF1A*; [22]), Ribosomal protein L13A (*RPL13A*; [23]), and Glucose-6-phosphate dehydrogenase (*G6PD*; [24]) to serve as internal normalization factors (Supplementary S1 Table). Normalization in this context adjusts for technical variation such as differences in RNA extraction efficiency, cDNA synthesis yield, and overall reaction efficiency [25]. Specifically, each target gene’s expression is measured relative to one or more stable reference genes, ensuring that the observed differences in mRNA abundance reflect true biological variation rather than technical artifacts. To evaluate the stability of these candidate housekeeping genes, we used RefFinder, which integrates four commonly used methods—Delta C_T_, BestKeeper, NormFinder, and geNorm. These algorithms compare variation in cycle threshold (C_T_) values across experimental conditions to identify the most stable reference gene(s). The stability ranking indicated that *RPL13A* (1.19) was the most stable assay, followed by *EF1A* (1.41), while *G6PD* (9.00) did not perform reliably across treatments. Consequently, G6PD was excluded from further analyses. Such variability in certain housekeeping genes is not unexpected, as many traditional reference genes can be downregulated under specific stressors (e.g., osmotic or thermal stress; [26]). For example, *G6PD* may fluctuate in response to oxidative stress or shifts in metabolic demands [27], making it unsuitable under experimental conditions. For the initial chip performance assessment, we used raw C_T_ values. The ΔC_T_ method was employed specifically for the 24-hour challenge experiment. Further details on verifying endogenous controls and excluding less stable genes—a recognized best practice in transcription studies [28]—are provided in the section describing the 24-hour challenge data.

The 25 target genes selected for osmoregulatory stress detection were primarily based on differential gene expression studies conducted under varying salinity conditions in salmonids. Osmoregulatory gene functions include ion transporters, osmolyte synthesis enzymes, and other proteins for maintaining osmotic balance [29,30]; we selected twelve genes within those functions ([31–38]; see Supplementary S1 Table). We also selected four genes associated with the general stress response ([39–42]; see Supplementary S1 Table) since osmoregulatory stress can activate other stress-related responses, such as the hypothalamic-pituitary-interrenal axis and cortisol production. We included five immune function genes as osmoregulatory stress can impact the immune system, affecting susceptibility to pathogens ([43–47]; see Supplementary S1 Table). Finally, four genes related to growth and metabolism were included ([48–50]; see Supplementary S1 Table), as stress can influence metabolic processes and growth rates, helping to identify markers of stress-induced growth inhibition. These 28 Taqman^TM^ qPCR gene transcription assays were designed to be printed on OpenArray® plates, resulting in the OSTP chip.

### 2.2. Chip design and optimization

The target candidate genes had been previously used for qPCR analyses by the GEN-FISH (the Genomic Network for Fish Identification, Stress and Health project; https://gen-fish.ca/) co-investigators, and all had shown measurable responses to stress in different studies (see details in [21]. The primers and probes for the assays were designed to target conserved regions within the selected gene coding regions (to ensure broad taxonomic application within the Salmonidae group) by aligning all available salmonid sequences for each gene using the NCBI sequence (http://www.ncbi.nlm.nih.gov/Genbank/) database. The goal was to develop qPCR assays that would work across a broad range of salmonid species as possible to maximize the utility of the OpenArray® OSTP chip for transcriptional profiling. More specifically, the Atlantic salmon ortholog (or orthologs from other salmonids) were searched for using BLASTn (NCBI) for sequence identification, alignments, phylogenetic tree building, and primer/probe design using Geneious Prime software (version 7.1.5; [51]). A search query of 500 was used. All sequences from the *Salmo*, *Oncorhynchus*, *Salvelinus*, and *Coregonus* spp., as well as *Esox lucius* (as a closely-related non-salmonid species) and *Danio rerio* (as a well-annotated model species) were selected and aligned using the MUSCLE aligner. The extracted sequences contained only the coding regions (CDS). An initial alignment including all these sequences was used to generate a phylogenetic tree using the Geneious Tree Builder with the default settings (i.e., Tamura-Nei, Neighbor-joining). The cluster of sequences that grouped with the Atlantic salmon ortholog (or other salmonid orthologs) were extracted. These target sequences were realigned and used to design primers and probes based on a consensus sequence. It is possible that some assays may amplify paralogs; however, because the assays were intended to function as biomarkers, their response to stress was the primary consideration. The functional gene categories are listed in Supplementary S1 Table. The primer and probe sequences used for assay design are listed in Supplementary S2 Table. The accession numbers and species used in the final alignments for primer/probe design are also listed in Supplementary S2 Table.

Primers (forward and reverse) were designed to be 16–22 bp, with three or fewer consecutive identical nucleotides and to generate amplicons less than 150 bp. The primers were designed with a target of 55% GC content (range: 45–65%) to ensure stable binding. Probes were designed using similar criteria to ensure gene sequence specificity and sensitivity but to function across multiple salmonid species effectively. The probes were designed with a melting temperature between 56–62°C to yield efficient detection for the OpenArray® Taqman^TM^ qPCR platform. All primers and Taqman^TM^ probes for the 25 candidate and three endogenous control gene assays were developed using Primer Express Software v3.0 (Thermo Fisher Scientific) for use on the OpenArray® chip platform. Both the primers and probes underwent *in silico* species testing via BLAST searches to confirm their broad applicability across salmonid species. The OSTP chip was printed using OpenArray® technology, which allows high throughput multiplexing of assays on a single chip, enhancing efficiency and consistency across samples.

### 2.3. OSTP chip assessment

To assess the performance of the OpenArray® OSTP chip among salmonid species, we applied it to eight diverse species. The eight species represent four genera: Arctic char (*Salvelinus alpinus*, AC), Brook Trout (*Salvelinus fontinalis*, BRKT), Lake Trout (*Salvelinus namaycush*, LT), Atlantic Salmon (*Salmo salar*, AS), Bloater (*Coregonus hoyi*, BLR), Lake Whitefish (*Coregonus clupeaformis*, LW), Chinook Salmon (*Oncorhynchus tshawytscha*, CS), Rainbow Trout (*Oncorhynchus mykiss*, RT). These species were selected due to their significance in sport and commercial fisheries, as well as their ecological and conservation importance.

Gill and liver tissues from three individuals from each species were provided by collaborators and shipped to the Great Lakes Institute for Environmental Research (GLIER), University of Windsor, for RNA extraction and cDNA synthesis. Different collaborators under the GEN-FISH project used the same Standard Operating Procedure (SOP) (e.g., euthanasia, sample preservation, etc.), making the samples are comparable, despite coming from different labs. The fish were all captive and sampled at rest, experiencing no obvious stress. RNA extractions from both gill and liver tissues used a TRIzol-based method (Invitrogen, Carlsbad, CA, USA) to provide high-quality RNA. Briefly, the tissue samples were homogenized using 1.0 mm glass beads and TRIzol, followed by phase separation with chloroform to isolate the RNA. Post-separation, the aqueous phase containing RNA was precipitated using isopropanol and washed with 70% ethanol to remove impurities. Selected RNA samples were used to estimate the RNA Integrity Number (RIN) on a 2100 Bioanalyzer to ensure the samples were suitable for subsequent analyses. The remaining RNA sample concentrations were measured using a NanoVue spectrophotometer (General Electric Company, Boston, MA, USA), and samples that fell below 500 ng/μL were re-extracted to ensure sufficient yield. DNase I (Ambion, Thermo Fisher Scientific, Waltham, MA, USA) was used to eliminate residual DNA. cDNA was then synthesized using the High-Capacity cDNA Reverse Transcription Kit (Applied Biosystems, Burlington, ON, Canada), following the manufacturer’s protocol. cDNA samples were stored at −20°C until they were used for qPCR.

qPCR assays were conducted using the Taqman^TM^ OpenArray® OSTP chip which had each assay printed in duplicate, resulting in six qPCR assays for each species for each gene (3 samples x 2 replicates). The QuantStudio 12K Flex PCR System (Thermo Fisher Scientific) was used to perform qPCR and generate initial C_T_ values through 40 cycles of amplification. Each 5.0 µL qPCR reaction mixture consisted of cDNA (1.2 µL per sample), ddH_2_O (1.3 µL) and Taqman^TM^ OpenArray® PCR Master Mix 2.5 µL (Applied Biosystems, Burlington, ON, Canada). The Taqman^TM^ OpenArray® chips were loaded using the OpenArray® AccuFill System to reduce inter-assay variation. The qPCR started with an initial denaturation step at 95°C for 10 minutes, followed by 40 cycles of denaturation at 95°C for 15 seconds and annealing/extension at 60°C for one minute. A total of 2 chips were used for 96 cDNA samples (3 samples x 2 biological replicates x 8 species x 2 tissues [gill and liver]). We used C_T_ values to assess the performance of the individual assays on the OSTP chip across all eight species. To determine expected qPCR assay performance variation among the eight selected salmonid species, the primer and probe sequences were aligned with each species’ corresponding gene sequences, and the number of nucleotide mismatches between the primer/probe and target species gene sequences were counted to quantify expected assay performance for each of the eight species across the 28 qPCR assays.

### 2.4. 24-hour saltwater challenge

To assess the OSTP chip’s performance under actual osmotic stress conditions, we performed a 24-hour saltwater challenge using Chinook salmon at the hatchery facilities of Yellow Island Aquaculture Ltd., a commercial salmon farm on Quadra Island, BC, Canada. The fish used in this experiment were 20 months post-fertilization with an average mass of 51.4 g (±0.3 SE) and were being reared in freshwater raceways. Two experimental groups were established: (1) Freshwater control (FW-FW), where fish were transferred between freshwater (FW) tanks, and (2) Saltwater treatment (FW-SW), where freshwater-reared fish were transferred to saltwater (SW) tanks (30 ppt). Fish were acclimated in freshwater tanks for 72 hours before the start of the experiment to recover from transport stress, after which they were transferred to four identical 200 L tanks (two freshwater and two saltwater tanks), the second transfer (after recovery from transport) constituted the acute stressor. Throughout the experiment, dissolved oxygen (DO) levels were maintained at an average of 9.58 mg/L, with an oxygen saturation of 88%. Sampling was conducted at the end of the 24-hour challenge. Six fish per treatment (three per replicate) were euthanized with an overdose of clove oil (CloveOilCorp; 0.4 mL/L clove oil in aerated hatchery water) – the fish were held in the anesthetic bath until they lost equilibrium, after which their spinal cords were severed before taking measurements and tissue samples. Liver and gill samples were collected within 3 minutes of euthanasia and were preserved in high salt buffer (see above) at 4°C for 24 hours before being transferred to −20°C for long-term storage. This experiment was designed to assess transcriptional responses to saltwater exposure, focusing on freshwater-reared Chinook salmon. All animals were treated following the guidelines provided by the Canadian Council on Animal Care during holding and experimentation, and approval was granted by the University of Windsor Animal Utilization Project Proposal (AUPP#23-06).

Gene transcription was quantified by calculating ΔC_T_ values. We then calculated ΔΔC_T_ values to assess the gene transcription response for the saltwater challenge relative to the mean control ΔC_T_ value. To display both up- and downregulation on a single scale, gene expression was expressed as ΔΔC_T_, where positive values indicate downregulation and negative values indicate upregulation.

### 2.5. Data analysis

All statistical analyses were conducted in R v4.5.0 [52]. For binary outcomes (amplification success), generalized linear mixed models (GLMMs) were fitted using the glmer() function in the lme4 package [53], specifying a binomial error distribution and a logit link. For continuous outcomes (C_T_ or log-transformed ΔC_T_ values), linear mixed models (LMMs) were fitted with the lmer() function in lme4. Tissue (gill vs. liver) and species were included as fixed effects. Target gene and species were modeled as random effects to account for variability across genes and species.

Model simplification followed a backward stepwise procedure: higher-order interaction and polynomial terms were tested first and removed if non-significant, followed by sequential removal of non-significant lower-order fixed effects. Random effects were assessed by likelihood ratio tests and retained only if they improved model fit. Collinearity among predictors was checked with variance inflation factors (VIFs). Model assumptions (normality, homoscedasticity) were evaluated using residual diagnostics (histograms, QQ plots, and Shapiro–Wilk tests).

For the 24-hour saltwater challenge, transcriptional responses (ΔΔC_T_) were analyzed relative to the FW-FW group at twenty-four hours, which served as the control condition. A two-way ANCOVA, with treatment (FW-FW vs. FW-SW) and tissue (gill vs. liver) as fixed factors, and body weight (g) included as a covariate, was used. Replicate tank was included as a random effect. Models were fitted with lmer() in lme4, and Type III sums of squares were obtained via Anova () in the car package [54]. Pairwise contrasts were estimated using emmeans, with Tukey adjustments [55]. False discovery rate (FDR) corrections were applied within functional gene categories (osmoregulation, stress response, immune function, growth & metabolism) using the Benjamini–Hochberg method. Statistical significance was determined at P < 0.05, and significant results are displayed by asterisks in the figures. All statistical analyses were run as described above.

Long format pairwise estimates for C_T_ differences are provided in Supplementary S3 Table; GLMM fixed and random effect estimates and odds ratios are provided in Supplementary S4 Table; Model visual diagnostics (histograms and QQ plots; Supplementary S1, S2 Figs) and Shapiro-Wilk tests.

## 3. Results

### 3.1. Primer-probe mismatch

We evaluated the match between primer/probe sequences across the eight target species to determine whether the observed species-specific effects on gene transcription could simply result from assay design (Table 1). The overall primer-probe mismatch rates across all genes showed that Chinook salmon (3.04%), followed by Arctic char (3.09%) and Atlantic salmon (3.1%) had the lowest mismatch rates, whereas, Bloater (5.72%) had the highest primer-probe mismatch.

**Table 1:** Proportion of mismatch nucleotides (%) for the forward and reverse primers and probe sequences across the 8 selected fish species based on NCBI gene alignments. The mismatch percentages are used to evaluate qPCR compatibility for each gene across species. A dash (“- “) indicates that no sequence was available on NCBI for that gene in that species.

Gene Category	Gene Name	*Salvelinus*			*Salmo*	*Coregonus*		*Oncorhynchus*	
		Arctic char	Brook trout	Lake trout	Atlantic salmon	Bloater	Lake whitefish	Rainbow trout	Chinook salmon
Endogenous control	*G6PD*	4.8%	6.0%	4.8%	5.1%	14.9%	10.5%	3.7%	3.7%
	*EF1A*	0.0%	0.0%	4.0%	0.0%	0.0%	0.0%	0.0%	8.7%
	*RPL13A*	0.0%	0.0%	0.0%	0.0%	0.0%	0.0%	0.0%	0.0%
Osmoregulation	*CFTR*	0.0%	6.6%	0.0%	0.0%	–	0.0%	4.5%	0.0%
	*ATP1A1*	6.8%	6.8%	1.8%	3.3%	8.4%	8.4%	10.5%	0.0%
	*ATP1B1*	1.8%	0.0%	6.6%	4.5%	–	0.0%	5.0%	6.7%
	*SLC2A11A*	0.0%	7.4%	7.4%	0.0%	5.7%	5.6%	0.0%	0.0%
	*SLC9A1B*	5.1%	5.1%	6.8%	4.8%	1.2%	1.6%	8.5%	5.7%
	*SLC9A2*	0.0%	3.6%	4.8%	8.8%	7.4%	1.8%	0%	0.0%
	*SLC9A3*	0.0%	0.0%	0.0%	0.0%	9.3%	2.9%	6.4%	0.0%
	*SLC12A1*	0.0%	1.6%	0.0%	0.0%	10.8%	8.9%	5.5%	0.0%
	*SLC12A2*	0.0%	6.3%	6.0%	0.0%	10.8%	2.1%	7.3%	1.6%
	*ATP6V1BA*	3.6%	1.8%	5.5%	6.8%	7.6%	9.1%	5.5%	5.7%
	*RHBG*	3.8%	1.6%	3.8%	5.5%	9.2%	9.0%	8.1%	1.5%
	*RHCG1*	8.6%	6.4%	6.4%	1.8%	6.4%	6.4%	2.1%	2.1%
Stress response	*GR1*	8.1%	2.8%	6.3%	3.5%	1.8%	1.8%	6.5%	2.7%
	*GR2*	1.0%	1.8%	4.4%	9.4%	7.4%	8.1%	0.0%	7.8%
	*HDS11B2*	0.0%	2.7%	2.7%	6.9%	6.4%	2.7%	0.3%	7.1%
	*HSP90BA*	3.3%	1.6%	3.3%	0.0%	7.6%	7.6%	1.6%	0.0%
Immune Function	*IL-1β*	9.3%	2.3%	4.7%	8.4%	1.1%	3.0%	0.0%	8.7%
	*IFNγ*	–	–	8.6%	1.4%	5.4%	0.0%	0.0%	0.0%
	*TNF-α*	3.3%	2.7%	5.6%	8.8%	–	10.6%	3.3%	3.5%
	*MX*	6.7%	3.4%	3.4%	2.8%	3.5%	3.5%	3.5%	8.9%
	*TAPBP*	7.6%	10.3%	8.5%	1.4%	8.8%	6.8%	3.2%	8.9%
Growth & Metabolism	*GHR*	0.0%	0.0%	0%	0.0%	0.0%	0.0%	0.0%	0.0%
	*IGF1*	7.9%	4.2%	4.2%	0.0%	4.3%	4.3%	0.0%	0.0%
	*IGFBP1*	0.0%	0.0%	0.0%	0.0%	0.0%	0.0%	0.0%	0.0%
	*IGFBP2*	1.7%	4.7%	2.4%	3.6%	5.1%	5.1%	3.6%	1.8%

### 3.2. Tissue and species-specific performances

Overall, gill-derived cDNA exhibited significantly better amplification success (83.0% of 1344 assays; 1116 successful amplifications; GLMM P < 0.001) and lower C_T_ values (mean C_T_ = 25.8) compared to liver-derived cDNA (63.4% of 1344 assays; 852 successful amplifications; mean C_T_ = 29.5; GLMM P < 0.01) (Tables 2 and 3). Assay success rates for gill and liver samples varied among the eight salmonid species (Table 2; Fig 1). Gill assays were generally more successful across species, with both Chinook salmon and Lake trout achieving the highest success rate of 96.4% (Table 2; Fig 1). For liver assays, Lake trout exhibited the highest success rate (78.6%), followed by Atlantic salmon (75%). In contrast, Arctic char showed the lowest liver assay success rate (42.9%), followed by Rainbow trout (46.4%).

**Table 2:** Tissue effect comparisons of mean C_T_ values between gill and liver tissues for eight salmonid species (and for all combined species) across 28 genes, with six replicates for both gill and liver cDNA, resulting in 168 assays per tissue per species. qPCR assay results are categorized as “Successful” (5–6 replicates), “Partial Success” (2–4 replicates), or “Failed” (0–1 replicates). Average C_T_ values are reported for successful assays only. P-values for tissue effects were calculated using a logistic generalized linear mixed model (GLMM) for amplification success and a linear mixed model (LMM) for C_T_ values, with adjustments for multiple comparisons using the Benjamini–Hochberg (FDR) method. Significance levels are (* P < 0.05, ** P < 0.01, *** P < 0.001) are shown in bold. Dashed lines (“- “) reflects the absence of data.

Species	Tissue	Failed	Partial Success	Successful	Proportion Successful	Avg_C_T_	P_value (Logistic GLMM)	P_value (LMM)	Total Assays
Arctic char	Gill	24	12	132	78.6%	26.4	0.15	0.11	168
	Liver	66	30	72	42.9%	28.8	0.22		168
Atlantic salmon	Gill	12	12	144	85.7%	24.1	**<0.05**	**<0.05**	168
	Liver	42	0	126	75.0%	29.6	0.06		168
Bloater	Gill	12	18	138	82.1%	26.1	**<0.05**	**<0.05**	168
	Liver	36	24	108	64.3%	28	0.07		168
Brook trout	Gill	6	30	132	78.6%	25.8	**<0.05**	**<0.05**	168
	Liver	24	30	114	67.9%	28.1	0.06		168
Chinook salmon	Gill	6	0	162	96.4%	25.1	**<0.01**	**<0.01**	168
	Liver	36	12	120	71.4%	30.4	**<0.05**		168
Lake whitefish	Gill	18	18	132	78.6%	26.2	0.1	0.1	168
	Liver	48	18	102	60.7%	29.7	0.18		168
Lake trout	Gill	6	0	162	96.4%	25.4	**<0.05**	**<0.05**	168
	Liver	18	18	132	78.6%	28.6	**<0.05**		168
Rainbow trout	Gill	30	24	114	67.9%	27.6	0.22	0.18	168
	Liver	72	18	78	46.4%	33.3	0.25		168
Gill Total	–	114	114	1116	83.0%	25.8	**<0.001**	**<0.001**	1344
Liver Total	–	342	150	852	63.4%	29.5	**<0.01**		1344
Overall Total	–	456	264	1968	73.20%	27.75	–	–	2688

**Table 3:** Mean C_T_ values from replicated Taqman^TM^ qPCR assays across 28 target genes using cDNA from gill and liver tissues of various salmonid species generated on the OpenArray® platform. Each value represents the mean C_T_ (cycle threshold) ± SEM, with the number of successful qPCR replicates (out of 6, 3 individuals replicated twice) in parentheses. Color coding demonstrates assay performance: red (0–1, failed), yellow (2–4, partial success), and green (5–6, successful amplification).

Gene Category	Gene Name	*Salvelinus*						*Salmo*		*Coregonus*				*Oncorhynchus*			
		Arctic char		Brook trout		Lake trout		Atlantic salmon		Bloater		Lake whitefish		Rainbow trout		Chinook salmon	
		Gill	Liver	Gill	Liver	Gill	Liver	Gill	Liver	Gill	Liver	Gill	Liver	Gill	Liver	Gill	Liver
Endogenous control	*G6PD*	26.4 ± 0.6 (6)	26.8 ± 0.6 (4)	27.6 ± 0.3 (6)	25.7 ± 0.4 (6)	25.4 ± 0.6 (6)	24.6 ± 0.8 (6)	24.1 ± 0.5 (6)	27.6 ± 0.7 (6)	26.1 ± 0.3 (6)	23.0 ± 0.3 (6)	26.0 ± 0.3 (6)	23.7 ± 0.3 (6)	29.0 ± 0.9 (6)	32.3 ± 0.1 (6)	25.1 ± 0.1 (6)	28.0 ± 0.1 (6)
	*EF1A*	25.6 ± 0.1 (6)	28.9 ± 0.5 (6)	25.8 ± 0.4 (6)	26.1 ± 0.1 (6)	25.6 ± 0.3 (6)	26.2 ± 0.5 (6)	24.8 ± 0.2 (6)	26.3 ± 0.4 (6)	25.5 ± 0.9 (6)	26.5 ± 0.1 (6)	25.9 ± 0.4 (6)	27.1 ± 0.4 (6)	27.4 ± 0.3 (6)	28.2 ± 0.4 (6)	24.5 ± 0.7 (6)	26.1 ± 0.9 (6)
	*RPL13A*	21.1 ± 0.2 (6)	25.9 ± 0.5 (6)	20.9 ± 0.7 (6)	19.3 ± 0.9 (6)	19.7 ± 0.7 (6)	19.7 ± 0.4 (6)	19.5 ± 0.6 (6)	19.6 ± 0.9 (6)	20.4 ± 0.1 (6)	20.3 ± 0.4 (6)	20.5 ± 0.2 (6)	21.8 ± 0.7 (6)	22.2 ± 0.1 (6)	21.7 ± 0.8 (6)	19.3 ± 0.5 (6)	18.8 ± 0.6 (6)
Osmoregulation	*CFTR*	27.2 ± 0.1 (6)	0 (0)	27.2 ± 0.6 (6)	29.7 ± 0.8 (6)	24.5 ± 0.1 (6)	0 (0)	24.6 ± 0.8 (6)	0 (0)	0 (0)	0 (0)	0 (0)	0 (0)	29.4 ± 0.4 (6)	0 (0)	23.0 ± 0.1 (6)	0 (0)
	*ATP1A1*	20.9 ± 0.3 (6)	0 (0)	20.5 ± 0.3 (6)	29.1 ± 0.2 (2)	22.1 ± 0.3 (6)	32.2 ± 0.7 (4)	19.3 ± 0.3 (6)	0 (0)	20.9 ± 0.5 (6)	28.9 ± 0.7 (4)	22.0 ± 0.1 (6)	22.0 ± 0.7 (2)	24.2 ± 0.6 (6)	0 (0)	20.4 ± 0.6 (6)	0 (0)
	*ATP1B1*	22.7 ± 0.8 (6)	27.6 ± 0.8 (6)	21.0 ± 0.4 (6)	21.3 ± 0.7 (6)	20.5 ± 0.2 (6)	21.9 ± 0.2 (6)	20.0 ± 0.5 (6)	22.2 ± 0.7 (6)	20.0 ± 0.4 (6)	22.6 ± 0.4 (6)	22.5 ± 0.7 (6)	24.3 ± 0.9 (6)	23.8 ± 0.3 (6)	25.5 ± 0.2 (6)	20.5 ± 0.2 (6)	22.3 ± 0.4 (6)
	*SLC2A11A*	0 (0)	0 (0)	32.9 ± 0.6 (4)	0 (0)	28.7 ± 0.5 (6)	0 (0)	0 (0)	0 (0)	31. 0.7 (6)	0 (0)	0 (0)	0 (0)	0 (0)	0 (0)	29.2 ± 0.5 (6)	0 (0)
	*SLC9A1B*	29.3 ± 0.7 (6)	21.6 ± 0.2 (2)	28.8 ± 0.8 (6)	29.7 ± 0.1 (6)	26.8 ± 0.9 (6)	29.6 ± 0.1 (6)	27.1 ± 0.7 (6)	31.1 ± 0.5 (6)	27.9 ± 0.4 (6)	31.2 ± 0.1 (6)	29.2 ± 0.4 (6)	31.3 ± 0.7 (6)	30.0 ± 0.5 (6)	29.6 ± 0.7 (2)	28.5 ± 0.6 (6)	30.4 ± 0.2 (6)
	*SLC9A2*	26.3 ± 0.7 (6)	0 (0)	27.5 ± 0.2 (6)	0 (0)	25.9 ± 0.3 (6)	0 (0)	25.4 ± 0.3 (6)	0 (0)	28.0 ± 0.3 (4)	0 (0)	28.4 ± 0.5 (6)	0 (0)	28.3 ± 0.2 (6)	0 (0)	25.0 ± 0.1 (6)	0 (0)
	*SLC9A3*	32.2 ± 0.4 (6)	0 (0)	21.9 ± 0.4 (6)	0 (0)	20.8 ± 0.1 (6)	31.6 ± 0.8 (2)	21.7 ± 0.1 (6)	0 (0)	31.2 ± 0.1 (6)	36.2 ± 0.3 (4)	34.6 ± 0.2 (6)	0 (0)	27.5 ± 0.1 (6)	0 (0)	21.5 ± 0.3 (6)	25.1 ± 0.3 (6)
	*SLC12A1*	29.9 ± 0.3 (4)	31.2 ± 0.5 (2)	30.2 ± 0.6 (4)	27.0 ± 0.3 (6)	28.0 ± 0.9 (6)	28.1 ± 0.2 (6)	28.6 ± 0.6 (6)	29.8 ± 0.1 (6)	30.2 ± 0.7 (6)	31.2 ± 0.6 (2)	30.1 ± 0.4 (2)	0 (0)	0 (0)	0 (0)	29.5 ± 0.5 (6)	26.5 ± 0.6 (6)
	*SLC12A2*	22.3 ± 0.9 (6)	29.9 ± 0.2 (6)	23.1 ± 0.9 (6)	27.8 ± 0.6 (6)	21.2 ± 0.3 (6)	27.0 ± 0.6 (6)	23.8 ± 0.9 (6)	28.8 ± 0.4 (6)	23.7 ± 0.5 (6)	30.7 ± 0.4 (6)	24.7 ± 0.8 (6)	31.2 ± 0.2 (6)	25.8 ± 0.6 (6)	30.0 ± 0.9 (4)	20.6 ± 0.8 (6)	26.7 ± 0.2 (6)
	*ATP6V1BA*	25.3 ± 0.3 (6)	28.4 ± 0.2 (6)	24.3 ± 0.5 (6)	24.5 ± 0.7 (6)	23.2 ± 0.7 (6)	24.0 ± 0.8 (6)	23.3 ± 0.3 (6)	25.2 ± 0.6 (6)	22.6 ± 0.9 (6)	23.8 ± 0.8 (6)	24.4 ± 0.1 (6)	26.9 ± 0.5 (6)	27.0 ± 0.4 (6)	27.8 ± 0.3 (6)	22.6 ± 0.9 (6)	24.1 ± 0.4 (6)
	*RHBG*	35.2 ± 0.5 (6)	0 (0)	23.0 + ± 0.2 (6)	27.2 ± 0.8 (6)	23.4 ± 0.1 (6)	27.6 ± 0.4 (6)	24.0 ± 0.1 (6)	28.6 ± 0.3 (6)	33.0 ± 0.4 (2)	0 (0)	34.5 ± 0.3 (6)	0 (0)	0 (0)	0 (0)	23.3 ± 0.3 (6)	26.9 ± 0.5 (6)
	*RHCG1*	21.8 ± 0.9 (6)	0 (0)	20.6 ± 0.1 (6)	30.5 ± 0.6 (2)	18.9 ± 0.2 (6)	29.5 ± 0.6 (6)	18.8 ± 0.4 (6)	0 (0)	18.6 ± 0.2 (6)	29.2 ± 0.5 (4)	19.9 ± 0.7 (6)	0 (0)	25.4 ± 0.6 (6)	0 (0)	18.7 ± 0.2 (6)	0 (0)
Stress response	*GR1*	26.4 ± 0.5 (6)	29.8 ± 0.3(4)	26.9 ± 0.7 (6)	27.3 ± 0.6 (6)	26.4 ± 0.4 (6)	28.1 ± 0.9 (6)	26.6 ± 0.1 (6)	28.3 ± 0.4 (6)	26.6 ± 0.8 (6)	27.1 ± 0.3 (6)	27.0 ± 0.2 (6)	29.2 ± 0.4 (6)	29.1 ± 0.7 (6)	28.8 ± 0.4 (6)	25.7 ± 0.8 (6)	26.8 ± 0.8 (6)
	*GR2*	26.6 ± 0.4 (6)	32.1 ± 0.5 (6)	26.4 ± 0.4 (6)	25.9 ± 0.3 (6)	25.1 ± 0.3 (6)	26.8 ± 0.4 (6)	25.7 ± 0.3 (6)	27.1 ± 0.5 (6)	25.7 ± 0.1 (6)	25.7 ± 0.1 (6)	26.4 ± 0.9 (6)	29.3 ± 0.2 (6)	28.7 ± 0.2 (6)	28.8 ± 0.8 (6)	24.7 ± 0.9 (6)	26.1 ± 0.4 (6)
	*HDS11B2*	25.3 ± 0.5 (6)	30.9 ± 0.8 (6)	27.5 ± 0.3 (6)	31.4 ± 0.7 (6)	24.9 ± 0.6 (6)	29.8 ± 0.1 (6)	26.8 ± 0.7 (6)	30.0 ± 0.1 (6)	26.8 ± 0.3 (6)	29.3 ± 0.8 (6)	27.8 ± 0.3 (6)	31.3 ± 0.7 (6)	26.3 ± 0.1 (6)	27.5 ± 0.3 (6)	25.1 ± 0.4 (6)	26.8 ± 0.9 (6)
	*HSP90BA*	26.1 ± 0.7 (6)	26.9 ± 0.1 (6)	26.9 ± 0.7 (6)	22.9 ± 0.3 (6)	26.6 ± 0.8 (6)	24.4 ± 0.5 (6)	26.2 ± 0.2 (6)	23.5 ± 0.6 (6)	26.2 ± 0.7 (6)	25.5 ± 0.3 (6)	26.9 ± 0.2 (6)	24.1 ± 0.2 (6)	30.5 ± 0.9 (6)	31.2 ± 0.1 (6)	26.4 ± 0.2 (6)	24.4 ± 0.1 (6)
Immune function	*IL-1β*	30.5 ± 0.1 (6)	0 (0)	29.2 ± 0.1 (6)	29.5 ± 0.3 (2)	26.9 ± 0.4 (6)	31.4 ± 0.5 (2)	27.8 ± 0.3 (4)	0 (0)	31.0 ± 0.2 (6)	0 (0)	30.2 ± 0.7 (4)	31.4 ± 0.8 (2)	29.6 ± 0.9 (2)	0 (0)	25.5 ± 0.7 (6)	30.9 ± 0.4 (4)
	*IFNγ*	34.1 ± 0.6 (2)	0 (0)	31.2 ± 0.4 (2)	33.1 ± 0.2 (2)	28.5 ± 0.7 (6)	29.6 ± 0.3 (6)	29.8 ± 0.2 (6)	30.1 ± 0.6 (6)	31.5 ± 0.2 (4)	0 (0)	32.5 ± 0.1 (6)	0 (0)	31.0 ± 0.3 (2)	0 (0)	25.7 ± 0.9 (6)	29.4 ± 0.2 (4)
	*TNF-α*	0 (0)	0 (0)	0 (0)	0 (0)	29.6 ± 0.3 (6)	31.2 ± 0.1 (6)	30.6 ± 0.4 (6)	32.4 ± 0.7 (6)	31.7 ± 0.8 (6)	36.4 ± 0.6 (2)	31.3 ± 0.7 (6)	32.6 ± 0.9 (2)	32.7 ± 0.5 (4)	0 (0)	28.2 ± 0.4 (6)	32.1 ± 0.7 (6)
	*MX*	28.1 ± 0.6 (6)	30.9 ± 0.2 (2)	28.2 ± 0.2 (6)	28.2 ± 0.7 (6)	24.7 ± 0.8 (6)	27.4 ± 0.5 (6)	25.6 ± 0.3 (6)	29.9 ± 0.4 (6)	25.7 ± 0.1 (6)	28.7 ± 0.4 (6)	25.0 ± 0.1 (6)	29.3 ± 0.1 (6)	30.2 ± 0.1 (6)	31.7 ± 0.3 (2)	23.1 ± 0.1 (6)	26.7 ± 0.5 (6)
	*TAPBP*	25.2 ± 0.7 (6)	27.5 ± 0.6 (4)	26.5 ± 0.7 (6)	26.5 ± 0.4 (6)	26.0 ± 0.4 (6)	28.2 ± 0.3 (6)	29.3 ± 0.1 (6)	27.8 ± 0.2 (6)	29.3 ± 0.4 (6)	32.1 ± 0.1 (6)	29.2 ± 0.7 (6)	32.4 ± 0.7 (6)	26.4 ± 0.9 (6)	28.3 ± 0.5 (6)	24.5 ± 0.8 (6)	27.9 ± 0.1 (6)
Growth & Metabolism	*GHR*	28.4 ± 0.5 (6)	29.8 ± 0.2 (6)	27.9 ± 0.1 (6)	25.1 ± 0.2 (6)	26.5 ± 0.6 (6)	24.2 ± 0.1 (6)	25.9 ± 0.5 (6)	24.1 ± 0.3 (6)	25.9 ± 0.1 (6)	23.1 ± 0.7 (6)	29.6 ± 0.9 (6)	27.6 ± 0.2 (6)	30.1 ± 0.3 (6)	26.2 ± 0.4 (6)	26.2 ± 0.7 (6)	23.5 ± 0.8 (6)
	*IGF1*	29.1 ± 0.3 (6)	25.7 ± 0.9 (6)	26.1 ± 0.2 (6)	23.4 ± 0.3 (6)	26.0 ± 0.1 (6)	19.8 ± 0.3 (6)	30.2 ± 0.8 (6)	22.2 ± 0.1 (6)	30.7 ± 0.3 (6)	28.2 ± 0.4 (6)	34.2 ± 0.6 (6)	28.9 ± 0.5 (6)	30.8 ± 0.2 (4)	23.5 ± 0.2 (6)	25.9 ± 0.1 (6)	22.2 ± 0.3 (6)
	*IGFBP1*	0 (0)	27.4 ± 0.8 (6)	28.5 ± 0.6 (2)	25.7 ± 0.5 (6)	32.1 ± 0.3 (6)	25.7 ± 0.6 (6)	0 (0)	21.4 ± 0.6 (6)	33.2 0.7 (4)	25.7 ± 0.6 (6)	0 (0)	28.9 ± 0.2 (6)	0 (0)	28.9 ± 0.4 (6)	28.5 ± 0.4 (6)	19.5 ± 0.6 (6)
	*IGFBP2*	0 (0)	0 (0)	30.4 ± 0.8 (4)	24.0 ± 0.2 (4)	0 (0)	29.3 ± 0.8 (6)	31.7 ± 0.4 (4)	23.6 ± 0.9 (6)	0 (0)	22.5 ± 0.8 (6)	31.8 ± 0.3 (2)	26.7 ± 0.8 (6)	0 (0)	0 (0)	0 (0)	0 (0)

**Fig 1:**
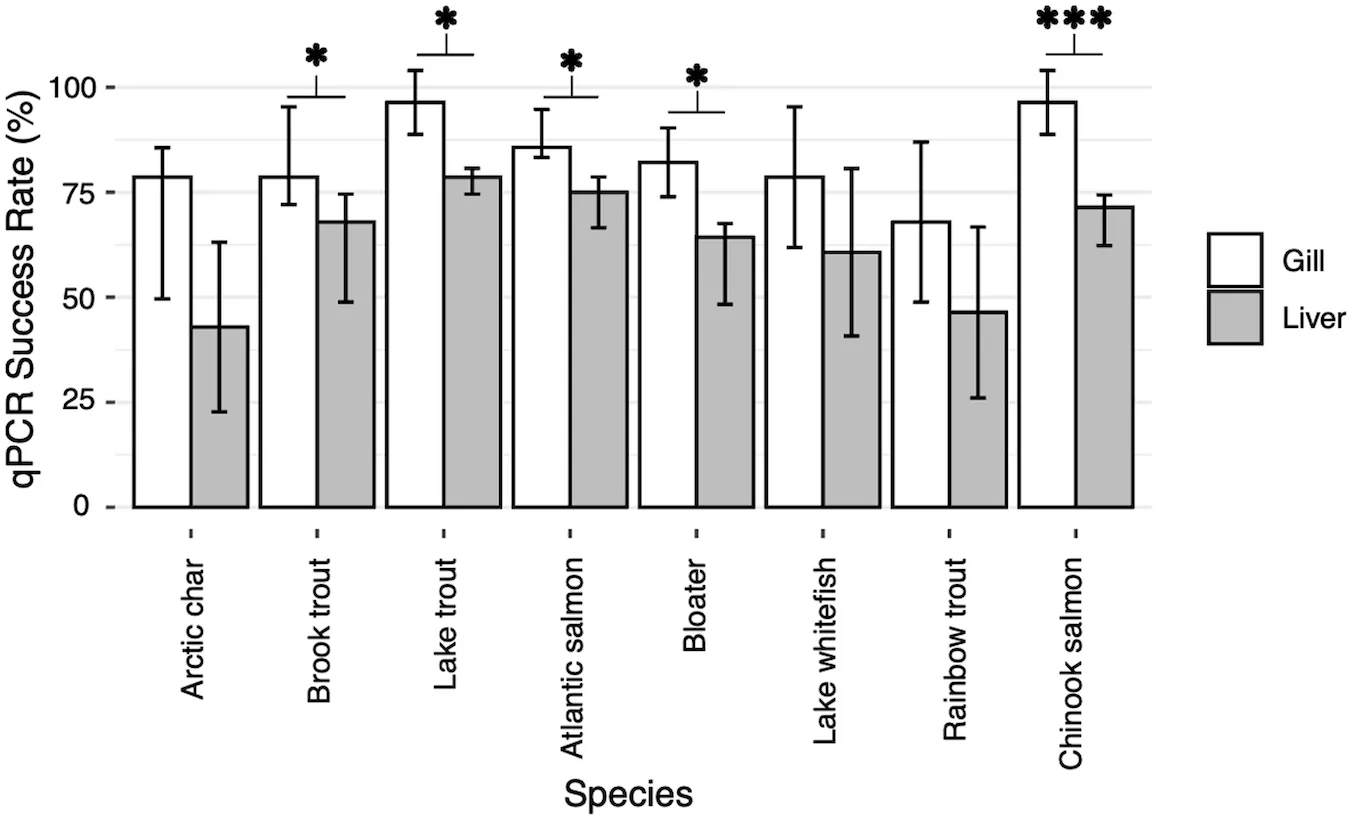
Comparison of qPCR success rates between gill and liver tissues across eight salmonid fish species. Comparison of qPCR success rates across eight fish species (Arctic char, Atlantic salmon, Bloater, Brook trout, Lake trout, Chinook salmon, Lake whitefish, and Rainbow trout) between two tissue types: gill (white bars) and liver (grey bars). The x-axis represents species, and the y-axis shows qPCR success rates expressed as the percentage of assays that produced a C_T_ value. Success was defined as the percentage of assays that yielded a C_T_ value out of a total of 2688 assays (8 species × 28 genes × 6 replicates × 2 tissues). Error bars represent the standard error of the mean (SEM) calculated from replicate success rates for each species and tissue. Significance levels are displayed above the bars: *P < 0.05, **P < 0.01, ***P < 0.001.

Across species, higher mean C_T_ values generally corresponded to fewer successful replicates, with partial success (2–4 replicates) or failed amplification (0–1 replicate) also appearing in the table (Tables 2 and 3). With gill cDNA, 10 osmoregulatory genes (*CFTR, SLC2A11A, SLC9A2, SLC9A3, SLC12A1, SLC12A2, ATP1A1, ATP1B1, RHBG,* and *RHCG1*) showed substantially lower C_T_ (≥2 units) in gill relative to liver, often also associated with higher amplification success in gill and partial or failed amplification in liver (Tables 2 and 3). Three immune genes (*IL-1β, TNF-α*, and *MX*) showed mean C_T_ values favoring gill and success rates exceeding 70% across species. These patterns were consistent across the osmoregulation and immune gene categories (Tables 2 and 3). However, liver cDNA showed higher transcript abundance (≥2 C_T_ difference) than gill cDNA for all four growth & metabolism genes (*GHR, IGF1, IGFBP1*, and *IGFBP2*), often with > 90% amplification success in liver tissue (Tables 2 and 3). In contrast, none of the osmoregulatory, immune, or stress-related targets or the endogenous control assays showed higher transcription in liver. We found no significant differences in the performance of the endogenous control gene assays between gill and liver (Tables 2 and 3).

### 3.3. Chinook salmon 24-hour saltwater challenge

The 24-hour saltwater challenge with Chinook salmon revealed distinct transcriptional responses in gill and liver tissues, with significant changes observed across osmoregulatory, stress, immune, and growth/metabolism gene categories. Relative transcription data, presented as ΔΔC_T_, highlighted tissue-specific dynamics in transcriptional activity. Additionally, ΔC_T_ values (calculated as the mean C_T_ of gill minus the mean C_T_ of liver) were used to compare average transcription levels between these tissues under experimental conditions, rather than reflecting baseline expression differences in unstressed fish.

In gill tissue, among osmoregulatory genes, *SLC12A2* showed significant upregulation in gene transcript abundance (Fig 2), whereas, *CFTR*, *SLC9A2*, and *ATP6V1BA* showed significant downregulation in gene transcript abundance. Other osmoregulatory genes exhibited no significant transcriptional differences. In the stress response category, *GR1* and *GR2* showed significant upregulation in gene transcript abundance (Fig 2). Immune-related and growth and metabolism genes did not exhibit significant transcriptional changes in gill tissue (Fig 2). In liver tissue, only *GHR* and *IGF1* exhibited significant upregulation among the growth and metabolism genes. No other genes among any other gene function category showed significant up- or downregulation in transcript abundance in liver tissue (Fig 2).

**Fig 2:**
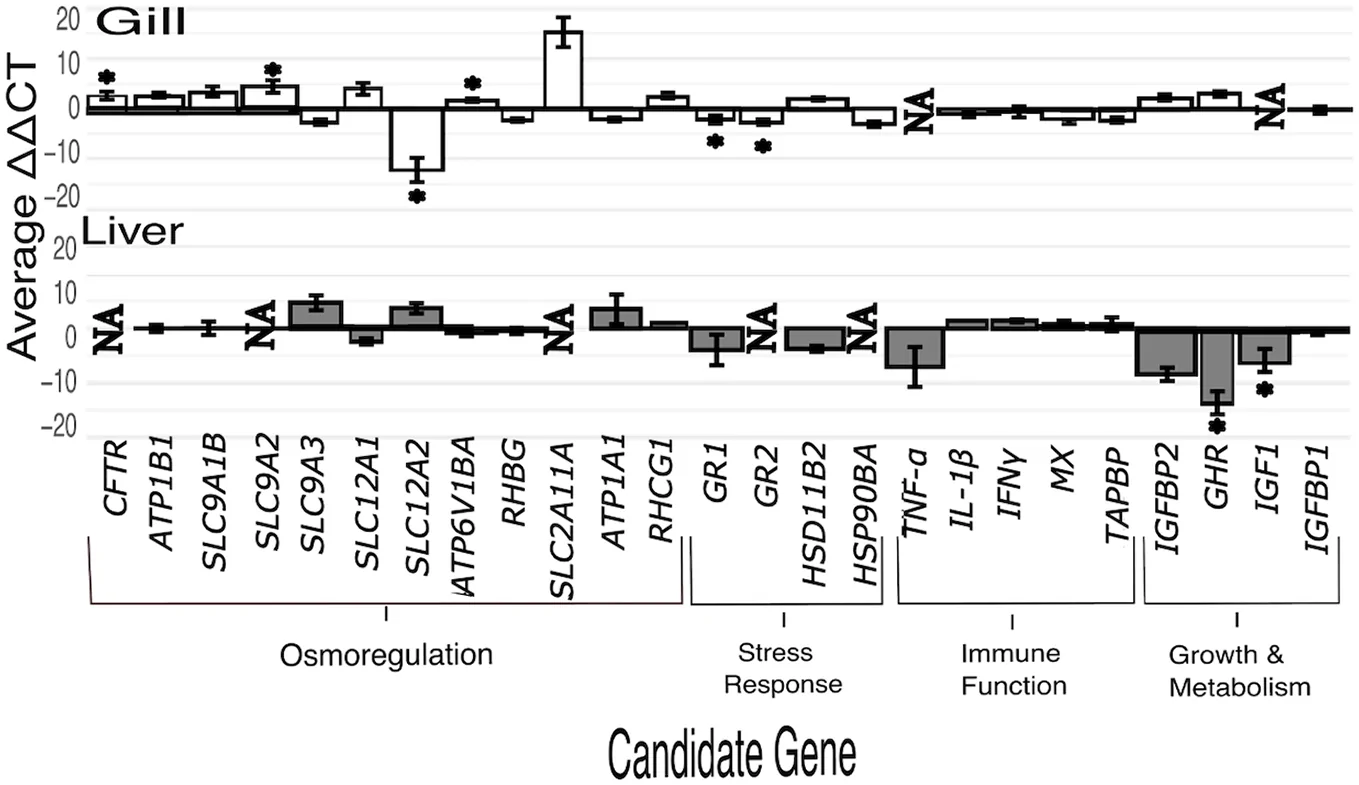
Relative transcriptional responses (ΔΔC_T_) in gill and liver tissues of Chinook salmon following a 24-h saltwater challenge. Bar graphs showing relative transcription response (displayed as ΔΔC_T_ values) in gill (white bars) and liver (grey bars) tissue after a 24-hour saltwater challenge in Chinook salmon. The 25 gene assays are categorized into osmoregulation (12 genes), stress response (four genes), immune function (five genes), and growth & metabolism (four genes). Bars represent the average ΔΔC_T_ values relative to control fish transcription, with error bars displaying standard error of the mean (SEM). Significant differences between tissues are indicated by an asterisk (*). Some gene assays did not provide data and are marked as not available (NA).

All genes across different biological function categories showed negative ΔC_T_ values (except for *SLC2A11A*, *SLC9A2*, and *SLC12A1* in the osmoregulatory gene category, and *IGF1*, *IGFBP1*, and *IGFBP2* in the growth and metabolism category), indicating that transcription levels were more highly expressed in gill than in liver tissue (Fig 3).

**Fig 3:**
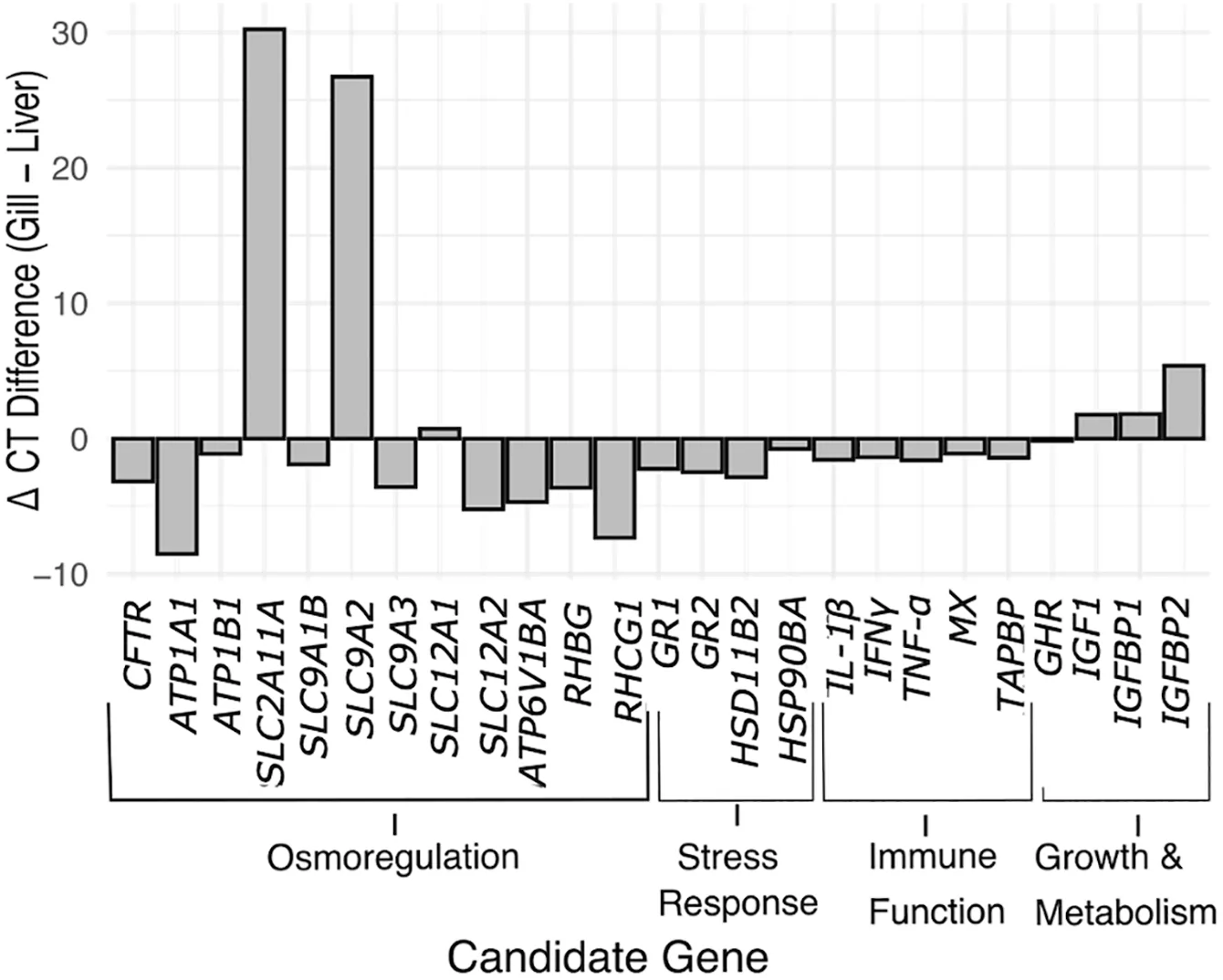
Differences in gene expression (ΔC_T_) between gill and liver tissues in Chinook salmon following a 24-h saltwater challenge. Bar graph showing the ΔC_T_ differences in gene expression between gill and liver tissues in Chinook salmon following a 24-hour saltwater challenge. The data presented here are ΔC_T_ values. The 25 gene assays are grouped into functional categories—osmoregulation (12 genes), stress response (four genes), immune function (five genes), and growth & metabolism (four genes). Positive ΔC_T_ values indicate higher relative expression in liver, while negative ΔC_T_ values indicate higher relative expression in gill. Bars represent the calculated ΔC_T_ values for each gene, with gene categories.

## 4. Discussion

The overall performance of the OSTP chip assays varied across tissues and species and generally, gill-derived cDNA performed better than liver-derived cDNA in both the eight at-rest species experiment and the 24-hour saltwater challenge. This is not surprising given that many of the assays were designed for osmoregulatory genes, which are often more actively expressed in the gill as it is a primary site for osmoregulation in fish. Liver cDNA samples exhibited reduced performance for assays targeting immune function and osmoregulation genes, although some assays within these functional categories worked well, depending on the species. Although the liver is known to be involved in fish immune function [56], the reduced success for some of our qPCR assays suggests that our specific gene assays may have limited applicability to liver tissue, although immune challenge applications should be explored. Overall, our study suggests that gill tissue can be used non-lethally and readily sampled, making this tissue particularly useful for conservation applications rather than using lethal liver tissue, which requires animal sacrifice [5,16].

Species-level differences in chip performance were also observed (e.g., Arctic char and Rainbow trout showed fewer successful qPCR amplifications, whereas Chinook salmon, Lake trout, and Atlantic salmon showed more), but no consistent genus-level effects were obvious. Sequence mismatch between primer/probe and target template sequences may have contributed to the variability in assay performance among the eight target species. Moreover, the qPCR amplification success rate among species might vary due to different lab protocols [19]; however, different collaborators under the GEN-FISH project used the same SOP/protocol to minimize handling effects (e.g., the method of euthanasia or sample preservation). These findings emphasize the importance of considering tissue- and species-specific variability in transcriptional profiling. While the OSTP chip has demonstrated significant utility, further refinement and expansion could enhance its applicability, improving performance for underperforming tissues and optimizing assay compatibility across diverse salmonid species.

Our study reveals that the qPCR assays exhibited significant tissue-specific transcriptional differences between gill and liver cDNAs across all eight species, emphasizing the specialized functions of these organs in the physiological response to stress. These findings align with a study on Atlantic salmon, Brown trout, Chinook salmon, Rainbow trout, Brook trout, Arctic char, Lake whitefish, and Bloater, which observed similar trends in stress- and osmoregulation-related gene expression in gill tissue across these same eight salmonid species [21]. Additionally, this cited study [21] observed lower amplification success for immune-related genes in liver and muscle tissues compared to gills [21]. In contrast, our study showed more consistent expression across tissues, which may reflect differences in the applied stressors or species-specific responses. However, the exclusive expression of *SLC2A11A* and *SLC9A2* in gills highlights their pivotal roles in ion transport and osmotic regulation, essential for maintaining ionic balance in aquatic environments [57,58]. Additionally, the elevated expression of immune genes in gill aligns with their function as frontline barriers against environmental pathogens, reinforcing localized immune responses [59].

Conversely, liver-derived cDNA demonstrated stronger transcription of metabolism and growth-related genes. These findings align with a study on the liver-specific expression of *IGFBP* paralogs in Atlantic salmon, which emphasizes their role in modulating *IGF* activity during stress and growth regulation [48]. It also emphasized the pivotal role of *IGFBP1* and *IGFBP2* in regulating *IGF* bioavailability under stress and catabolic conditions. Similarly, temperature-dependent modulation of *GHR* and *IGFBP* genes has been demonstrated in Atlantic salmon, with increased *GHR* transcription in muscle at elevated temperatures (17–19°C), suggesting lipolysis to meet energy demands [60]. These findings align with a study on Atlantic salmon, which reported increased *IGFBP1b* expression at intermediate temperatures (15–17°C), highlighting the liver’s role in regulating *IGF* bioavailability under environmental challenges [60]. In contrast, our study observed consistently high *GHR* expression in the liver, reinforcing its systemic role in energy regulation and metabolic adaptation. Prolonged thermal stress was also shown to reduce *IGF1* and *IGF2* mRNA levels in both liver and muscle, reflecting a shift from growth to energy mobilization [48]. Together, these results emphasize the interconnected roles of *GHR* and *IGFBP*-related genes in liver tissue in coordinating growth and metabolism in response to environmental stress.

Interestingly, a subset of genes involved in cellular homeostasis and stress response, such as *GR1*, *GR2*, *HSD11B2*, and *HSP90BA*, maintained comparable expression levels in both gill and liver tissues. These genes exhibited consistent transcription levels across tissues, emphasizing their essential role in maintaining cellular stability and mitigating damage from environmental stressors. This pattern aligns with findings from a comparative study on eight species of salmonids, where similar expression profiles were observed for these genes, further supporting their function in cellular stress management [21]. This consistent pattern of expression suggests a fundamental role in maintaining cellular stability and mitigating damage from environmental stressors across different tissues [19]. The ability of the OSTP chip to capture gene transcription in both gill and liver tissue suggests its potential for broad applications in other tissue types, including mucus swabs, representing an emerging non-invasive method for gene transcription profiling [21]. By facilitating the detection of both tissue-specific and cross-tissue transcriptional responses, the OSTP chip enables a comprehensive evaluation of gene transcription patterns.

While the OSTP chip exhibited generally robust performance across tissues and species in our broad at-rest assessment, its practical application is important for showcasing its utility as a transcriptional profiling tool. To assess its effectiveness in detecting molecular responses under physiological stress, we performed a 24-hour saltwater challenge with Chinook salmon and used the OSTP chip to profile their transcriptional responses to acute salinity stress. As observed in the multi-species analysis, liver-derived cDNA showed lower qPCR success compared to gill-derived cDNA, with only 20 of 25 assays yielding successful amplifications in liver versus 23 of 25 in gill. The saltwater challenge elicited significant responses in osmoregulatory and stress-related genes in gill tissue, reflecting an adaptive response to maintain ion balance under acute salinity stress. Overall, for saltwater-challenged Chinook salmon, 20 and 23 assays were successful in liver and gill, compared to 18 and 22 successful assays, respectively, in resting fish. By comparing the resting and challenged outcomes, our findings highlight the distinct transcriptional roles of gill and liver tissues in responding to acute salinity stress. The study further emphasizes the utility of the OSTP chip in detecting molecular responses to physiological challenges, with key insights into the roles of osmoregulatory, stress, and metabolic pathways during short-term stress responses.

Transcriptional profiling using the OSTP chip described here provides a framework for characterizing how salmonid fishes cope with osmoregulatory challenges. While our OSTP chip was conceived for applications in captive-reared fish to provide a tool to improve rearing practices and enhance fish performance [61], it also has value for wild populations, where it can be used to monitor responses to changing environmental conditions [62]. As part of the GEN-FISH project, we also designed tissues- and stressor-specific chips (see [63–66]) to foster the assessment of the health of salmonid fish under changing environments. Multi-scale controlled laboratory and field validation of these custom chips will be useful approaches for real-world applications to key environmental stressors. By targeting key genes involved in osmoregulation, stress, immune, and growth/metabolism, the OSTP chip allows extrapolation to possible impacts on survival, growth, and performance related to osmoregulatory stress. Although our OSTP chip was designed for, and performs better in, gill cDNA, it is clear that the chip can also provide valuable data using other tissues, which will be useful to measure stress responses in the wild. Furthermore, the OSTP chip could be used to characterize the remarkable osmoregulatory plasticity of salmonid species relative to other teleosts, providing valuable insights into their ability to adapt to diverse salinity conditions. Thus, the OSTP chip will be useful for aquaculture practices by fostering better husbandry as well as providing a valuable tool for future studies of the osmoregulatory response of salmonid fishes to environmental stress.

## Supporting information

S1 Table List of candidate genes and their function. (PDF)

S2 Table Primer and probe sequences. (PDF)

S3 Table Pairwise comparisons of mean C_T_ values among eight salmonid species. (PDF)

S4 Table Effects of species and tissues on amplification success. (PDF)

S1 Fig Histogram of model residuals. (PDF)

S2 Fig QQ plot of residuals. (PDF)
