# Supplementary material for: Gene transcriptional profiling of osmoregulatory stress response in salmonids: Development and assessment of high-throughput multispecies assays
Source: PLoS One. 2026 Oct 9;21(10):e0360513. doi: 10.1371/journal.pone.0360513 (PMC13652239; doi:10.1371/journal.pone.0360513)
Supplement: Supplementary file 1 [file pone.0360513.s001.pdf]

Supplementary S1 Table: List of candidate gene loci selected for the osmoregulatory stress-response transcriptional profiling (OSTP) chip, with putative function. The candidate genes were chosen to have important roles in survival, growth, performance and are associated with osmoregulation stress response.

Gene name	Function
**Endogenous control**	
Elongation factor 1-alpha (*EF1A*)	Catalyzes GTP-dependent binding of aminoacyl-tRNA to the ribosome's acceptor site, playing a key role in translation [22].
Ribosomal protein L13A (*RPL13A*)	Part of the GAIT complex, it mediates selective translation inhibition in response to interferon-gamma during inflammation [23].
Glucose-6-phosphate dehydrogenase (*G6PD*)	Serves as a crucial electron donor for defense against oxidizing agents and in reductive biosynthesis [24].
**Osmoregulation**	
Cystic fibrosis transmembrane conductance regulator (*CFTR*)	Complex protein found on the surface membrane of cells in a wide variety of tissues where it functions as a regulated chloride ion channel [31].
Sodium potassium ATPase alpha subunit (*ATP1A1*)	Considered as the FW isoform involved in ion absorption. Freshwater Isoform is predominantly involved in ion-uptake [32].
Sodium potassium ATPase subunit beta-1 (*ATP1B1*)	SW-isoform driving ion-secretion is important for ion regulatory competence during salinity transfer [32].
Solute carrier family 9 member A3, tandem duplicate 2 (NHE3) (*SLC9A3*)	Supports Na^+ uptake, as indicated by reduced whole-body Na^+ uptake and H^+-ATPase-powered electrogenic uptake in H^+-ATPase-rich cells [33].
Solute carrier family 9 member A2 (NHE2) (*SLC9A2*)	Predominantly localized at the basolateral side of cells at the base of the lamellae, indicating a role in chloride transport [33].
Solute carrier family 2 member 11a (*SLC2A11A*)	Rapidly adjusts glucose levels in response to environmental changes, with elevated plasma glucose indicating stress and impacting energy metabolism [37].
ATPase, H+ transporting, lysosomal V1 subunit β, member a (*ATP6V1BA*)	Plays a role in the acidification of intracellular compartments and hence its lack of function induces apoptosis in those tissues in which it is normally expressed [34].

Solute carrier family 12 member 2 (Na-K-CL- cotransporter isoform 1) (*SLC12A2*)	Function is modulated by intracellular electrolyte concentration, which impacts thermodynamic stability of the interaction between intracellular proteins [36].
Solute carrier family 9-member 1b (*SLC9A1B*)	Carries out an influx of chloride and efflux of bicarbonate [35].
Solute carrier family 12 member 1 (*SLC12A1*)	Absorbs Cl^- via the Na^+-Cl^- co-transporter on the apical membrane and extrudes it into the blood through the CLC-2c channel on the basolateral side of gill epithelial cells [36].
Rh family, B glycoprotein (*RHBG*)	Facilitates ammonia secretion in distal epithelial cells, with expression localized to the basolateral plasma membrane [38].
Rh type C glycoprotein (*RHCG1*)	Restoration of ammonia excretion in the face of elevated external ammonia [38].
**Stress response**	
Glucocorticoid receptor 1 (*GR1*)	Binds glucocorticoid hormones in the liver, muscle, and vasculature, influencing metabolism and cardiovascular function [40].
Glucocorticoid receptor 2 (*GR2*)	Displays distinct DNA-binding domain structures in teleosts, indicating receptor diversity [39].
11β-hydroxysteroid dehydrogenase 2 (*HSD11B2*)	Key enzyme in the cortisol signaling pathway, catalyzing cortisol conversion and controlling the cortisone switch [41].
Heat shock protein 90 (inducible) (*HSP90BA*)	Essential molecular chaperone involved in homeostatic control during normal and stress conditions [42].
**Immune Function**	
Interleukin 1β (*IL-1β*)	Crucial for host response to pathogens; can worsen damage in chronic diseases and acute injuries [43].
Interferon γ (*IFNγ*)	Protects salmon cells from infectious pancreatic necrosis virus (IPNV) by reducing viral titers and inhibiting viral protein synthesis [44].
Tumor necrosis factor α (*TNF-α*)	Inflammatory cytokine produced by macrophages/monocytes, mediating signaling events leading to necrosis or apoptosis; important for infection and cancer resistance [45].

Tapasin (*TAPBP*)	Glycoprotein essential for stabilizing MHC class I complexes and regulating peptide loading [46].
Mx protein (*MX*)	Induced by double-stranded RNA (dsRNA) in fish cells, primarily through type I interferon (IFN) activation in response to synthetic dsRNA stimulation [47].
**Growth and Metabolism**	
Insulin-like growth factor 1 (*IGF-I*)	Suppresses growth hormone (GH) secretion from the pituitary and hypothalamus in a negative feedback loop [48].
Growth hormone receptor (*GHR*)	Activation by growth hormone triggers tyrosine kinase activity, leading to rapid phosphorylation of proteins and induction of early-response genes [49].
Insulin-like growth factor binding protein 1 (*IGFBP1*)	Inhibits IGF action by preventing its interaction with receptors [50].
Insulin-like growth factor binding protein 2 (*IGFBP2*)	Inhibits IGF-I action, with levels increasing under catabolic conditions; remains stable without clear hormonal control [50].
