# Supplementary material for: Gene transcriptional profiling of osmoregulatory stress response in salmonids: Development and assessment of high-throughput multispecies assays
Source: PLoS One. 2026 Oct 9;21(10):e0360513. doi: 10.1371/journal.pone.0360513 (PMC13652239; doi:10.1371/journal.pone.0360513)
Supplement: Supplementary file 2 [file pone.0360513.s002.pdf]

Supplementary S2 Table: Osmoregulatory stress-response transcriptional profiling (OSTP) nanofluidic chip candidate genes used for Taqman™ qPCR assays of gene transcription on the OpenArray® platform. The candidate genes are arranged by putative function, and gene name (symbol), forward and reverse primer and probe sequences, amplicon size, accession numbers (a unique identifier for the specific DNA or protein sequence used in primer design) and species used in the final alignments for primer design are listed.

Gene Function	Gene Name	Forward/Reverse Primers & Probe sequence	Amplicon size (bp)	Species used in the final alignments for primer design	Accession Number
Reference Genes	Glucose-6-phosphate 1-dehydrogenase (*G6PD*)	F: GAGTGCCTTCTCCAACCTCCA<br>R: CAGCCCTTGGTACTCATGCAG<br>P: CTACCTCGCCCTGCC	147	*S. salar*<br>*S. trutta*<br>*O. keta*<br>*O. mykiss*<br>*O. tshawytscha*<br>*O. gorbuscha*<br>*O. kisutch*<br>*S. namaycush*	NM_001141724<br>XM_029723525<br>XM_035738273<br>XM_021566625<br>XM_024384803<br>XM_046365258<br>XM_020459406<br>XM_039011009
	Elongation factor 1 alpha (*EF1A*)	F: GAAGCTTGAGGACAACCCCA<br>R: GAAGCTCTCCACACACATGGG<br>P: CCGCCATCATCGTCAT	92	*S. salar*<br>*S. trutta*<br>*O. keta*<br>*O. mykiss*<br>*O. nerka*<br>*O. tshawytscha*<br>*O. kisutch*<br>*S. alpinus*<br>*S. namaycush*<br>*C. clupeaformis*	NM_001141909<br>XM_029735646<br>XM_035795162<br>XM_021561980<br>XM_029658054<br>XM_024396037<br>XM_031793751<br>XM_024146326<br>XM_038971575<br>XM_041903035
	Ribosomal protein L13a (*RPL13A*)	F: CACTGGAGAGGCTGAAGGTGT<br>R: GTGGGCTTCAGACGGACAAT<br>P: CATGGTCGTACCTGCT	103	*O. masou*<br>*S. salar*<br>*S. trutta*<br>*O. keta*<br>*O. mykiss*<br>*O. nerka*<br>*O. tshawytscha*<br>*O. kisutch*	EU325865<br>XM_014128281<br>XM_029730684<br>XM_035742484<br>XM_021574753<br>XM_029688610<br>XM_024437735<br>XM_020488314

				S. alpinus<br>*S. namaycush*<br>*C. clupeaformis*	XM_023994886<br>XM_039009297<br>XM_041855521
Osmoregulation	Cystic fibrosis transmembrane conductance regulator (Cl-channel) (*CFTR*)	F: CAGGCAGAAAAGAGCAGGGA<br>R: TCTCCATCACCTCCTCCCAG<br>P: AGTCGTCGCCTGGC	107	*S. salar*<br>*S. trutta*<br>*O. keta*<br>*O. mykiss*<br>*O. tshawytscha*<br>*O. nerka*<br>*S. namaycush*<br>*S. alpinus*<br>*C. clupeaformis*	NM_001123533<br>XM_029697084<br>XM_035798038<br>XM_036979278<br>XM_042323330<br>XM_029634857<br>XM_039000654<br>XM_023970781<br>XM_041836795
	Sodium-potassium ATPase alpha subunit (*ATP1A1*)	F: TGTGTCCGTCTTCTTCGGTG<br>R: CAGTAGCCAGGAGACCCTCTG<br>P: GCTATGGATGGCTGCC	126	*O. mykiss*<br>*S. salar*<br>*S. trutta*<br>*O. tshawytscha*<br>*O. kisutch*<br>*O. masou*<br>*S. namaycush*	NM_001124461<br>XM_045722983<br>XM_029748747<br>XM_042316867<br>XM_031818113<br>AB573640<br>XM_038981298
	Sodium-potassium ATPase subunit beta-1 (*ATP1B1*)	F: GGATGCTTGGCTGGGATTT<br>R: CTCTGTCCTGGTAGGTGGGTTT<br>P: CAGGCCCTGCTGCT	83	*S. salar*<br>*S. trutta*<br>*O. mykiss*<br>*O. tshawytscha*<br>*O. kisutch*<br>*S. alpinus*<br>*S. namaycush*<br>*C. clupeaformis*	NM_001252362<br>XM_029708332<br>XM_036979818<br>XM_042322829<br>XM_031822295<br>XM_024142989<br>XM_039003039<br>XM_041840719
	Solute carrier family 2-member 11a (*SLC2A11A*)	F: GTTCGCTGACCGTAATGTGC<br>R: ATGGAGTCGTTGCCACAGAG<br>P: CAGCAGCGCCATGC	84	*S. salar*<br>*O. keta*<br>*O. mykiss*<br>*O. tshawytscha*<br>*O. kisutch*<br>*O. gorbuscha*	NM_001141793<br>XM_035778241<br>XM_036950146<br>XM_024374110<br>XM_020486753<br>XM_046311987

				S. namaycush<br>*S. alpinus*<br>*C. clupeaformis*	XM_038970559<br>XM_071380116<br>XM_041889167
	Solute carrier family 9 member A1B (*SLC9A1B*)	F: CTTCATCTTCCTGGGCGTGT<br>R: GACGAAGGTCCAGTTCCAGG<br>P: CACGGTGGCAGGGC	61	*O. mykiss*<br>*S. salar*<br>*O. keta*<br>*O. tshawytscha*<br>*O. gorbuscha*<br>*O. kisutch*<br>*S. namaycush*	NM_001124695<br>XM_014142630<br>XM_035757656<br>XM_024395440<br>XM_046361737<br>XM_020500134<br>XM_038972353
	Solute carrier family 9 member 2 (*SLC9A2*)	F: CATCTCAGCTGTGGACCCAG<br>R: TCGTTGAACAGGCACTCTCC<br>P: CCGTGCTCAGCGTGT	102	*O. mykiss*<br>*S. salar*<br>*S. trutta*<br>*O. tshawytscha*<br>*O. nerka*<br>*O. kisutch*<br>*S. alpinus*<br>*S. namaycush*<br>*C. clupeaformis*	NM_001130994<br>XM_014174391<br>XM_029712099<br>XM_024410130<br>XM_029646675<br>XM_020499911<br>XM_024010456<br>XM_039006593<br>XM_041843915
	Solute carrier family 9 member A3, tandem duplicate 2 (*SLC9A3*)	F: ATCTTCGTCCTGGGCTACCTC<br>R: ACAGCAGACCCCACAGAATG<br>P: AGCCGAGATGCTCT	90	*O. mykiss*<br>*S. salar*<br>*S. trutta*<br>*O. keta*<br>*O. tshawytscha*<br>*O. gorbuscha*<br>*O. nerka*<br>*O. kisutch*<br>*S. alpinus*	NM_001160482<br>XM_014141359<br>XM_029712099<br>XM_035793565<br>XM_024396240<br>XM_046362575<br>XM_029663816<br>XM_020500716<br>XM_023975187
	Solute carrier family 12 member 1 (*SLC12A1*)	F: CCTGGTTGGACATCCTCACC<br>R: GAAGGTGAGGACGCTCTTGTG<br>P: ACCTCCCTCCCACCAT	77	*O. tshawytscha*<br>*S. salar*<br>*S. trutta*<br>*O. mykiss*<br>*O. kisutch*	XM_042316068<br>NM_001141520<br>XM_029720918<br>XM_036981072<br>XM_031820808

				S. namaycush	XM_038987605
	Solute carrier family 12 member 2 (Na-K-Cl cotransporter isoform 1) (*SLC12A2*)	F: CGTTTTGCATCGCTCTAGCC<br>R: AGCATAGGAGGCCAGGAAGA<br>P: TCGCTCCCATCATCT	89	*S. salar*<br>*S. trutta*<br>*O. keta*<br>*O. mykiss*<br>*O. tshawytscha*<br>*O. nerka*<br>*O. gorbuscha*<br>*S. alpinus*<br>*S. namaycush*	NM_001123683<br>XM_029710291<br>XM_031802486<br>XM_036976792<br>XM_042302639<br>XM_029647200<br>XM_046290487<br>XM_023984575<br>XM_038970029
	ATPase, H+ transporting, lysosomal, V1 subunit B, member a (*ATP6V1BA*)	F: AGAAGACGGCCTGTGAGTTCA<br>R: GTCCCAGCATGTCCTCAGACA<br>P: CGGTGACATCCTGCGC	66	*S. salar*<br>*O. mykiss*<br>*O. tshawytscha*<br>*S. namaycush*	NM_001141746<br>XM_021609923<br>XM_024424712<br>XM_038960498
	Rh family, B glycoprotein (*RHBG*)	F: CACAGCCACCGTTCTGATCTC<br>R: TGATGAGGAGCTGGACAGGAC<br>P: CGTTCGGGGCAGTGC	68	*S. salar*<br>*S. trutta*<br>*O. keta*<br>*O. tshawytscha*<br>*O. nerka*<br>*O. gorbuscha*<br>*O. kisutch*<br>*S. alpinus*	NM_001141124<br>XM_029756072<br>XM_035774348<br>XM_024377707<br>XM_029677301<br>XM_046297897<br>XM_020455545<br>XM_023975561
	Rh type C glycoprotein (*RHCG1*)	F: TTCCTGTGGATGTTCTGGCC<br>R: AGGTGTTGATGACTGCCCTG<br>P: CATGGAGACGGTCAGC	82	*O. mykiss*<br>*S. salar*<br>*S. trutta*<br>*O. keta*<br>*O. tshawytscha*<br>*O. gorbuscha*<br>*O. nerka*<br>*O. kisutch*<br>*S. alpinus*	NM_001124523<br>XM_014175744<br>XM_029721355<br>XM_035787123<br>XM_024440305<br>XM_046345896<br>XM_029639391<br>XM_031812239<br>XM_024002622

Stress response	Glucocorticoid receptor 1 (*GR1)*	F: GACTCTGCTGCAGTGTTCCTG<br>R: CACTGGTCTGCCATGTAGGG<br>P: CCTTCGGCCTGGGC	150	*O. mykiss,*<br>*S. salar*<br>*O. tshawytscha*<br>*O. kisutch*<br>*S. alpinus*	NM_001124482<br>XM_014198677<br>XM_042303411<br>XM_031797047<br>XM_024137659
	Glucocorti-coid receptor 2 (*GR2*)	F: ATGGCAGACCAGTGTGAACAGAT<br>R: GAGCAGCAGCAGAACCTTCAT<br>P: AGACTGCAGGTGTCTCA	103	*O. mykiss*<br>*S. salar*<br>*S. trutta*<br>*O. tshawytscha*<br>*O. nerka*<br>*O. kisutch*<br>*S. alpinus*	AY495372<br>XM_014198677<br>XM_029771923<br>XM_024409462<br>XM_029655477<br>XM_031789624<br>XM_024137659
	11β-hydroxysteroid dehydrogenase 2 (*HSD11B2*)	F: CTGTCTAGCAGCGTACGGAGC<br>R: ATGGTGGACACTTTGACCCC<br>P: CCTGTTCATCAACACACT	99	*O. mykiss*<br>*S. salar*<br>*S. trutta*<br>*O. nerka*<br>*O. kisutch*<br>*O. tshawyscha*<br>*C. clupeaformis*	NM_001124218<br>XM_014186204<br>XM_029768326<br>XM_029651578<br>XM_031812548<br>XM_024410377<br>XM_041859559
	Heat shock protein 90 (constitutive) (*HSP90BA*)	F: GAGGTGGAGGAGGACGAGTACA<br>R: GCTGTGAAGTGGATGTGGGA<br>P: TCTCCAGGGACACAGAC	86	*S. salar*<br>*S. trutta*<br>*O. keta*<br>*O. mykiss*<br>*O. nerka*<br>*O. tshawytscha*<br>*O. kisutch*<br>*S. alpinus*<br>*C. clupeaformis*	XM_014208111<br>XM_029761271<br>XM_035784415<br>XM_021577266<br>XM_029665926<br>XM_024416091<br>XM_020491013<br>XM_023966868<br>XM_041853504
	Interleukin 1β (*IL-1β*)	F: ACCTGTCCTGCTCCAAATCG<br>R: TGCTGGCTGATGGACTTCAG<br>P: CCCACCCTGCACCT	88	*S. salar*<br>*S. trutta*<br>*O. keta*<br>*O. mykiss*<br>*O. nerka*	NM_001123582<br>XM_029716234<br>XM_035786315<br>NM_001124347<br>XM_029638479

Immune Function				*O. tshawytscha*<br>*O. kisutch*<br>*S. alpinus*<br>*S. namaycush*<br>*C. clupeaformis*	XM_024418276<br>XM_020475860<br>XM_024002415<br>XM_039003225<br>XM_041901577
	Interferon γ (*IFNγ*)	F: GGAAGGCTCTGTCCGAGTTC<br>R: CTCTTCCTGCGGTTGTCCTT<br>P: CCTCCAAACTGGCC	91	*S. salar*<br>*S. trutta*<br>*O. mykiss*<br>*O. tshawytscha*<br>*O. namaycush*<br>*O. kisutch*<br>*C. clupeaformis*	NM_001171804<br>XM_029743588<br>FM864345<br>XM_042314919<br>XM_038995696<br>XM_031819098<br>XM_041873105
	Tumor necrosis factor α (*TNF-α*)	F: AGTACCAGACGCTGCTCAACTC<br>R: TTTTCCTTCACTCGCAGCCT<br>P: TCTGTACGCACCGTGTGT	77	*S. salar*<br>*S. trutta*<br>*O. keta*<br>*O. mykiss*<br>*O. tshawytscha*<br>*O. gorbuscha*<br>*O. kisutch*<br>*S. alpinus*<br>*S. namaycush*	NM_001123617<br>XM_029734485<br>XM_035767308<br>HE798146<br>XM_024407165<br>XM_046360750<br>XM_020468340<br>XM_024139369<br>XM_038972209
	Mx protein (*MX*)	F: GGAGGAGATTGAGGACCCCT<br>R: ATCACTGATACCCACCCCCA<br>P: TGAAGCCCAGGATGAA	88	*S. salar*<br>*S. trutta*<br>*O. keta*<br>*O. mykiss*<br>*O. nerka*<br>*O. tshawytscha*<br>*O. gorbuscha*<br>*O. kisutch*<br>*S. alpinus*<br>*S. namaycush*	NM_001123690<br>XM_029774982<br>XM_035777514<br>XM_021569609<br>XM_029683585<br>XM_024415945<br>XM_046313204<br>XM_020491468<br>XM_023993827<br>XM_039009004
	Tapasin (*TAPBP*)	F: ACGGCAAGACTGACCGATTT<br>R: CACTTCAGCTTCCTGCAGGAT	111	*S. salar*<br>*S. trutta*	NM_001123605<br>XM_029731732

		P: AGCGGGGCTAGACTT		*O. keta*<br>*O. mykiss*<br>*O. nerka*<br>*O. tshawytscha*<br>*O. gorbuscha*<br>*O. kisutch*<br>*S. alpinus*<br>*S. namaycush*<br>*C. clupeaformis*	XM_035795285<br>NM_001124553<br>DQ092324<br>XM_024441639<br>XM_046326692<br>XM_020505550<br>XM_023976454<br>XM_038966528<br>XM_041903149
Growth and Metabolism	Growth hormone receptor *(GHR)*	F: TAACCGGGAGCCACTTTGAC<br>R: ATTGACCTCACGGTACTGCACC<br>P: TGAGCTGGGAGCCG	110	*S. salar*<br>*S. trutta*<br>*O. keta*<br>*O. mykiss*<br>*O. nerka*<br>*O. tshawytscha*<br>*O. gorbuscha*<br>*O. kisutch*<br>*O. masou*<br>*S. alpinus*<br>*S. namaycush*<br>*C. clupeaformis*	NM_001123594<br>XM_029769265<br>XM_035772405<br>NM_001124731<br>XM_029620369<br>XM_024397386<br>XM_046317034<br>AF403540<br>AB071216<br>XM_023987611<br>XM_038964896<br>XM_041850797
	Insulin-like growth factor 1 (*IGF1*)	F: TTCAAGAGTGCGATGTGCTGT<br>R: CGCCGAAGTCAGGGTTAGG<br>P: ACACCCTCTCACTGCT	79	*S. salar*<br>*S. trutta*<br>*O. keta*<br>*O. mykiss*<br>*O. nerka*<br>*O. tshawytscha*<br>*O. gorbuscha*<br>*O. kisutch*<br>*S. alpinus*<br>*S. namaycush*<br>*C. clupeaformis*	NM_001123623<br>XM_029742642<br>XM_035748910<br>XM_021577176<br>XM_029665859<br>XM_024372796<br>XM_046297130<br>XM_031797471<br>XM_024014490<br>XM_038977529<br>XM_045214404

	Insulin-like growth factor binding protein 1 (*IGFBP1)*	F: AGAGCATCAAGGCCAAGGTC<br>R: CATGCAGCTCAATGTGGCAG<br>P: ACTGGTGGAACAGGGT	75	*S. salar*<br>*S. trutta*<br>*O. mykiss*<br>*O. keta*<br>*O. tshawytscha*<br>*O. nerka*<br>*O. gorbuscha*<br>*O. kisutch*<br>*S. alpinus*	NM_001279137<br>XM_029750591<br>NM_001124561<br>XM_035736623<br>XM_024420224<br>XM_029687052<br>XM_046351830<br>XM_020492902<br>XM_023997977
	Insulin-like growth factor binding protein 2 (*IGFBP2)*	F: CCTTCACGGGTGTCCAGATC<br>R: CTCCTCCACATACTGGCTGC<br>P: CCACGAAGGTGCGGG	74	*S. salar*<br>*S. trutta*<br>*O. keta*<br>*O. mykiss*<br>*O. nerka*<br>*O. gorbuscha*<br>*O. kisutch*<br>*S. alpinus*<br>*S. namaycush*	NM_001279160<br>XM_029703049<br>XM_035774215<br>XM_021579985<br>XM_029635193<br>XM_046368859<br>XM_020462099<br>XM_023980724<br>XM_039014863
