# Supplementary material for: Gene transcriptional profiling of osmoregulatory stress response in salmonids: Development and assessment of high-throughput multispecies assays
Source: PLoS One. 2026 Oct 9;21(10):e0360513. doi: 10.1371/journal.pone.0360513 (PMC13652239; doi:10.1371/journal.pone.0360513)
Supplement: Supplementary file 3 [file pone.0360513.s003.pdf]

Supplementary S3 Table: Pairwise comparisons of mean $C_T$ values among eight salmonid species, assessed using a linear mixed model with Species as a fixed effect and Target Gene as a random effect. Estimates represent differences in mean $C_T$ values between species (negative values indicate lower $C_T$ higher transcript abundance, in the first-listed species). Standard errors (SE), t-ratios, and p-values are provided. P-values were adjusted for multiple testing using the Benjamini–Hochberg false discovery rate (FDR) method. Degrees of freedom were constant across all contrasts (df = 413) and are omitted for clarity.

| contrast | estimate | SE | t.ratio | p.value |
|---|---|---|---|---|
| Arctic Char - Atlantic Salmon | -1.681714286 | 1.735801427 | -0.968840248 | 0.518298546 |
| Arctic Char - Bloater | -3.294071429 | 1.735801427 | -1.897723655 | 0.272671412 |
| Arctic Char - Brook Trout | -3.983071429 | 1.735801427 | -2.29465846 | 0.150874746 |
| Arctic Char - Chinook salmon | -1.875178571 | 1.735801427 | -1.080295558 | 0.518298546 |
| Arctic Char - Lake trout | -4.074392857 | 1.735801427 | -2.347268987 | 0.150874746 |
| Arctic Char - Lake Whitefish | -2.283678571 | 1.735801427 | -1.315633537 | 0.443431222 |
| Arctic Char - Rainbow Trout | 0.559892857 | 1.735801427 | 0.322555823 | 0.836858265 |
| Atlantic Salmon - Bloater | -1.612357143 | 1.735801427 | -0.928883407 | 0.520935698 |
| Atlantic Salmon - Brook Trout | -2.301357143 | 1.735801427 | -1.325818211 | 0.443431222 |
| Atlantic Salmon - Chinook salmon | -0.193464286 | 1.735801427 | -0.11145531 | 0.94506165 |
| Atlantic Salmon - Lake trout | -2.392678571 | 1.735801427 | -1.378428739 | 0.443431222 |
| Atlantic Salmon - Lake Whitefish | -0.601964286 | 1.735801427 | -0.346793289 | 0.836858265 |
| Atlantic Salmon - Rainbow Trout | 2.241607143 | 1.735801427 | 1.291396071 | 0.443431222 |
| Bloater - Brook Trout | -0.689 | 1.735801427 | -0.396934804 | 0.836858265 |
| Bloater - Chinook salmon | 1.418892857 | 1.735801427 | 0.817428097 | 0.579817184 |
| Bloater - Lake trout | -0.780321429 | 1.735801427 | -0.449545332 | 0.831439822 |
| Bloater - Lake Whitefish | 1.010392857 | 1.735801427 | 0.582090118 | 0.747764787 |
| Bloater - Rainbow Trout | 3.853964286 | 1.735801427 | 2.220279478 | 0.150874746 |
| Brook Trout - Chinook salmon | 2.107892857 | 1.735801427 | 1.214362901 | 0.450606135 |
| Brook Trout - Lake trout | -0.091321429 | 1.735801427 | -0.052610527 | 0.958067666 |
| Brook Trout - Lake Whitefish | 1.699392857 | 1.735801427 | 0.979024922 | 0.518298546 |
| Brook Trout - Rainbow Trout | 4.542964286 | 1.735801427 | 2.617214282 | 0.128667656 |
| Chinook salmon - Lake trout | -2.199214286 | 1.735801427 | -1.266973429 | 0.443431222 |
| Chinook salmon - Lake Whitefish | -0.4085 | 1.735801427 | -0.235337979 | 0.876683301 |

| | | | | |
|---|---|---|---|---|
| Chinook salmon - Rainbow Trout | 2.435071429 | 1.735801427 | 1.402851381 | 0.443431222 |
| Lake trout - Lake Whitefish | 1.790714286 | 1.735801427 | 1.03163545 | 0.518298546 |
| Lake trout - Rainbow Trout | 4.634285714 | 1.735801427 | 2.66982481 | 0.128667656 |
| Lake Whitefish - Rainbow Trout | 2.843571429 | 1.735801427 | 1.63818936 | 0.408575128 |
