# Supplementary material for: Gene transcriptional profiling of osmoregulatory stress response in salmonids: Development and assessment of high-throughput multispecies assays
Source: PLoS One. 2026 Oct 9;21(10):e0360513. doi: 10.1371/journal.pone.0360513 (PMC13652239; doi:10.1371/journal.pone.0360513)
Supplement: Supplementary file 4 [file pone.0360513.s004.pdf]

Supplementary S4 Table: Fixed effects from the logistic generalized linear mixed model (GLMM) predicting amplification success among eight salmonid species by tissue. The dependent variable was amplification success (binary: 1 = success, 0 = failure), with Tissue (Gill vs. Liver) as the primary fixed effect and Species as a secondary fixed effect. Odds Ratios indicate the relative likelihood of amplification success. Statistical significance is shown as *** ($P < 0.001$), ** ($P < 0.01$), * ($P < 0.05$).

**Term**	**Estimate**	**Std. Error**	**z-value**	**p-value**	**Signif.**	**Odds Ratio**
(Intercept)	4.2192	1.1343	3.72	0.0002	***	67.98
Tissue (Liver)	-2.3125	0.4141	-5.584	2.35E-08	***	0.1
Species (Atlantic Salmon)	1.226	0.6709	1.828	0.0676	.	3.41
Species (Bloater)	1.4809	0.6862	2.158	0.0309	*	4.4
Species (Brook Trout)	2.3116	0.7555	3.06	0.00222	**	10.08
Species (Chinook Salmon)	1.7316	0.7038	2.46	0.0139	*	5.65
Species (Lake Trout)	2.6774	0.7981	3.355	0.0008	***	14.54
Species (Lake Whitefish)	0.7371	0.6473	1.139	0.255		2.09
Species (Rainbow Trout)	-0.4593	0.6205	-0.74	0.459		0.63
