# Supplementary material for: Gene transcriptional profiling of osmoregulatory stress response in salmonids: Development and assessment of high-throughput multispecies assays
Source: PLoS One. 2026 Oct 9;21(10):e0360513. doi: 10.1371/journal.pone.0360513 (PMC13652239; doi:10.1371/journal.pone.0360513)
Supplement: Supplementary file 5 [file pone.0360513.s005.pdf]

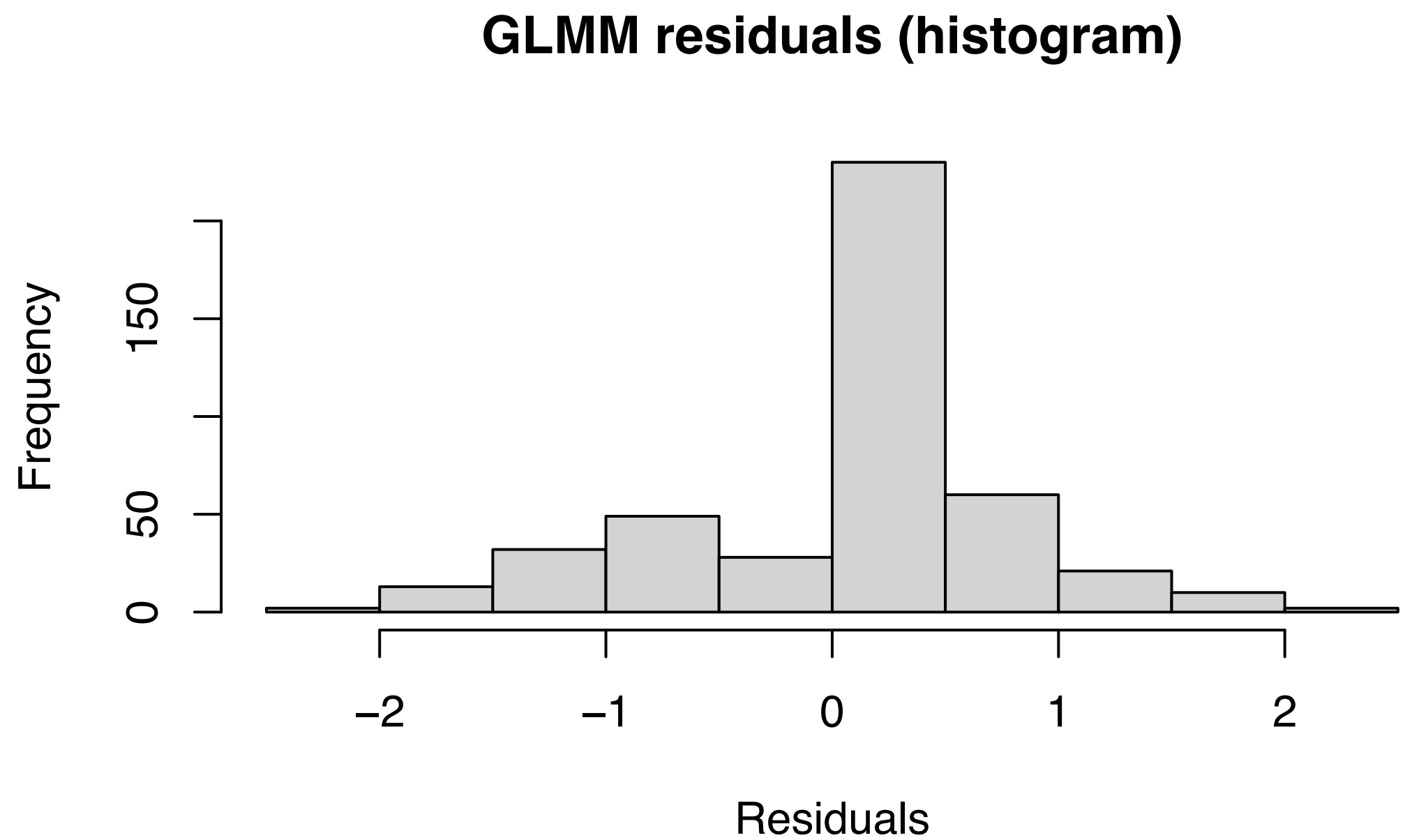

Supplementary S1 Figure. Histogram of model residuals from the generalized linear mixed model (GLMM) assessing amplification success across species and tissues. The distribution shows departures from normality, which is expected for binomially distributed outcomes. Residual inspection confirmed no major violations affecting overall model fit.
