# Supplementary material for: Gene transcriptional profiling of osmoregulatory stress response in salmonids: Development and assessment of high-throughput multispecies assays
Source: PLoS One. 2026 Oct 9;21(10):e0360513. doi: 10.1371/journal.pone.0360513 (PMC13652239; doi:10.1371/journal.pone.0360513)
Supplement: Supplementary file 6 [file pone.0360513.s006.pdf]

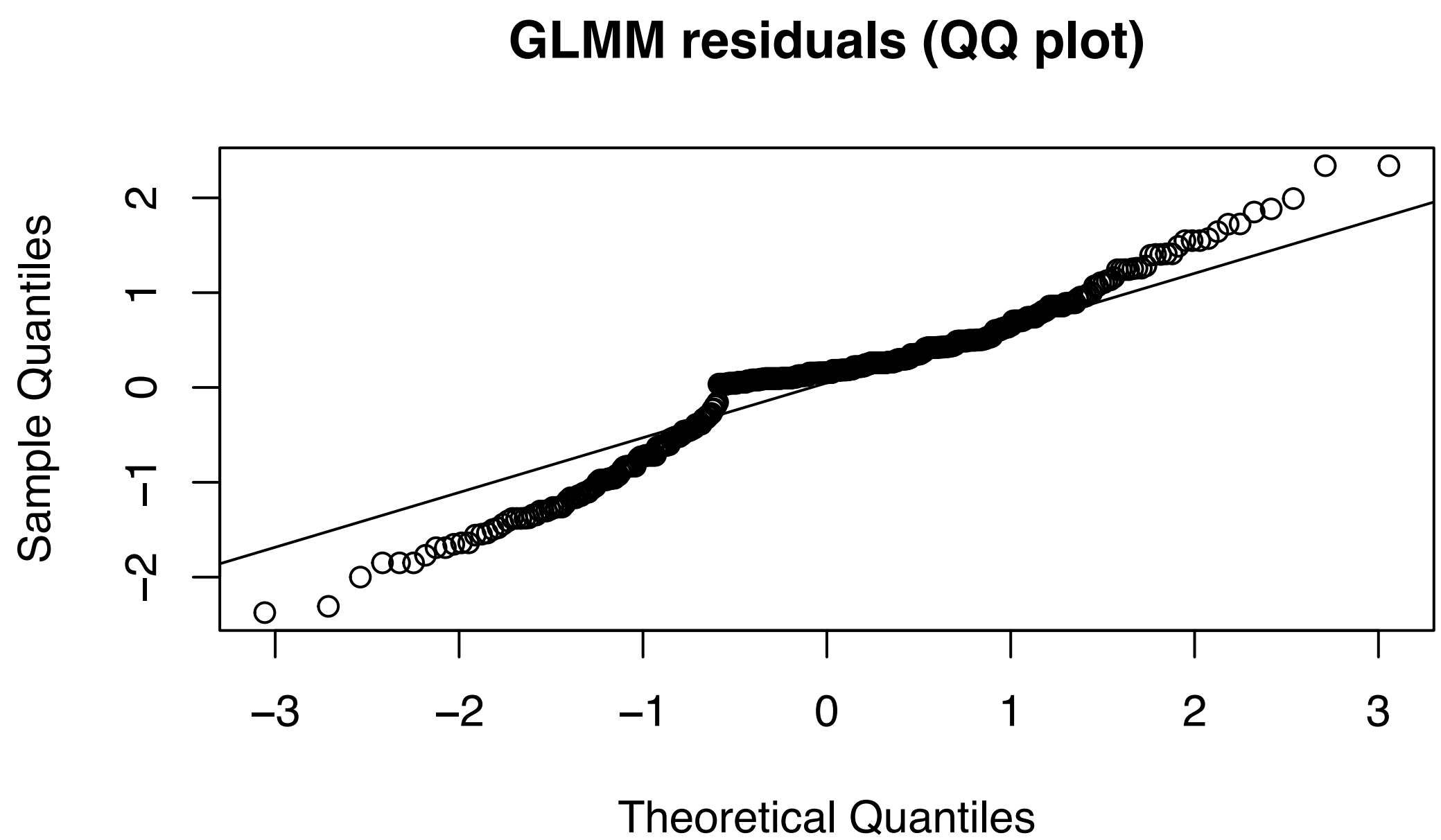

Supplementary S2 Figure. Quantile-quantile (QQ) plot of residuals from the GLMM for amplification success. Deviations from the 1:1 line indicate residuals are not normally distributed (Shapiro-Wilk test, $P < 0.001$). This result is consistent with the binomial error distribution of the model and does not compromise inference from fixed effects.
